# ALG-2 interacting protein-X (Alix) is essential for clathrin-independent endocytosis and signaling

**DOI:** 10.1038/srep26986

**Published:** 2016-05-31

**Authors:** Vincent Mercier, Marine H. Laporte, Olivier Destaing, Béatrice Blot, Cédric M. Blouin, Karin Pernet-Gallay, Christine Chatellard, Yasmina Saoudi, Corinne Albiges-Rizo, Christophe Lamaze, Sandrine Fraboulet, Anne Petiot, Rémy Sadoul

**Affiliations:** 1 Institut National de la Santé et de la Recherche Médicale (INSERM), Unité 1216, F-38042 Grenoble, France; 2 Université Grenoble Alpes, Institut des Neurosciences, F-38042 Grenoble, France; 3 INSERM U1209, Grenoble, F-38042, France; 4 Université Grenoble Alpes, Institut Albert Bonniot, F-38000 Grenoble, France; 5 CNRS UMR 5309, F-38000 Grenoble, France; 6 Institut Curie, PSL Research University, Membrane Dynamics and Mechanics of Intracellular Signaling Laboratory, Paris, France; 7 INSERM, U1143, Paris, France; 8 CNRS, UMR 3666, Paris, France

## Abstract

The molecular mechanisms and the biological functions of clathrin independent endocytosis (CIE) remain largely elusive. Alix (ALG-2 interacting protein X), has been assigned roles in membrane deformation and fission both in endosomes and at the plasma membrane. Using Alix ko cells, we show for the first time that Alix regulates fluid phase endocytosis and internalization of cargoes entering cells via CIE, but has no apparent effect on clathrin mediated endocytosis or downstream endosomal trafficking. We show that Alix acts with endophilin-A to promote CIE of cholera toxin and to regulate cell migration. We also found that Alix is required for fast endocytosis and downstream signaling of the interleukin-2 receptor giving a first indication that CIE is necessary for activation of at least some surface receptors. In addition to characterizing a new function for Alix, our results highlight Alix ko cells as a unique tool to unravel the biological consequences of CIE.

The plasma membrane of all eukaryotic cells undergoes constant renewal through repeated cycles of endocytosis and exocytosis. During endocytosis, cell surface proteins and lipids are internalized forming vesicular carriers which then merge with early endosomes, a process central to the regulation of nutrient uptake, cell surface receptor signaling, plasma membrane remodeling, cellular mobility and synaptic vesicle recycling^1^. Most of these processes rely on clathrin-mediated endocytosis (CME) based on the clathrin machinery for shaping endocytic vesicles. However alternative pathways, collectively referred to as clathrin-independent endocytosis (CIE), also occur at the plasma membrane, although the molecular mechanisms leading to membrane bending and fission, as well as the biological significance of these pathways have yet to be clarified^2^.

An important advance in defining the molecular players involved in CIE came from two recent reports demonstrating that endophilin-A controls CIE of activated receptors including those for epidermal growth factor (EGF) and interleukin-2 (IL2), and that this pathway is hijacked by shiga- or cholera bacterial toxins^3^,^4^. The three endophilin-A isoforms (A1, A2, A3) contain a Src Homology 3 (SH3) domain binding to both dynamin and synaptojanin^5^ and a N-BAR (Bin/amphiphysin/Rvs) domain capable of sensing and generating membrane curvature^6^. At synapses, Endophilins-A are known to be involved in both CME^7^,^8^,^9^ and CIE^10^.

We have shown previously that besides dynamin and synaptojanin, a major interacting partner of endophilin-A is Alix (ALG-2-interacting protein X), first identified through its calcium-dependent binding to the penta-EF-hand protein ALG-2 (apoptosis-linked gene 2)^11^,^12^. Alix is a 95 kD cytoplasmic protein with multiple interacting partners. The N-terminal Bro1-like domain^13^, binds the endosome-resident lipid, lysobisphosphatidic acid (LBPA)^14^,^15^ and the charged multivesicular body protein 4B (CHMP4B) component of the endosomal sorting complex III required for transport (ESCRT-III)^16^, while the C-terminal long proline-rich domain (PRD) binds endophilin-A^12^, and also contains distinct interaction sites for ALG-2, the tumor suppressor gene 101 (Tsg-101) component of ESCRT-I, and Cbl Interacting protein-85 (CIN85)^17^. The central region of Alix consists of a V-shaped domain composed of two triple-helical bundles which mediate protein dimerization^18^. Alix is ubiquitously expressed and has been involved in numerous biological processes including programmed neuronal death^19^,^20^,^21^, virus egress^22^, cytokinesis^23^,^24^, cell spreading^25^ and membrane repair^26^,^27^. Until today, most of these activities have been linked with Alix capacity to bind and recruit proteins of the ESCRT complexes involved in membrane bending and fission. These complexes act in outward vesiculation, thus forming vesicles in endosomes and at the cell surface^28^,^29^, whereas other Alix interactors, CD2AP/CIN85 and endophilin-A, act in the opposite way to promote membrane invaginations during ligand-dependent receptor endocytosis^30^,^31^,^32^. The function of Alix binding to these latter proteins remains unknown.

Here, we have used mouse embryonic fibroblasts (MEFs) from Alix homozygous knock-out mice (Alix ko) to explore further the role of Alix in the endosomal pathway. We found that fluid phase endocytosis and internalization of several ligands were impaired in Alix ko cells, even though endosomal morphology and downstream intracellular trafficking were apparently normal. Interestingly, impaired endocytosis affected only CIE but not CME. We also demonstrate that, in the case of cholera toxin (CTx) CIE, the function of Alix is strictly dependent on its capacity to bind to endophilin-A. Finally, we provide the first demonstration that cargo endocytosis through the Alix/endophilin-A pathway is required for cell migration and IL2 Receptor (IL2R) signaling.

## Results

### Loss of Alix delays EGFR degradation

**Figure 1:**
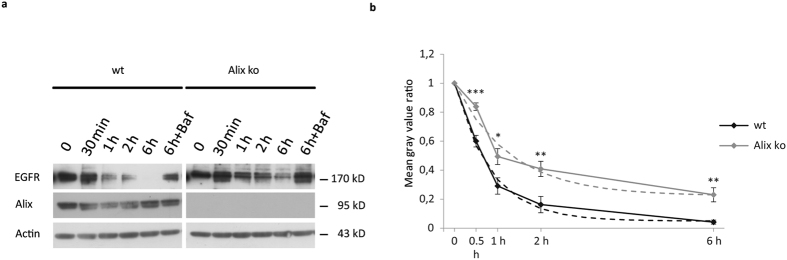
EGFR degradation is delayed in Alix ko cells. (**a**,**b**) Serum-starved wt or Alix ko MEFs were incubated with 100 ng/ml EGF at 37 °C for the indicated times. EGFR was revealed by immunoblotting (**a**) and band intensities estimated (**b**) using ImageJ software. EGFR intensities were normalised to actin. Graph represent the ratio between values at each time point and at t = 0 (n = 6; *P < 0.05, **P < 0.01, ***P < 0.001, two-tailed Student’s t-test). Dashed lines indicate degradation curves fitted with single exponential functions.

To investigate the role of Alix in endocytosis, we used MEFs derived from Alix ko mice obtained in our laboratory, in which expression of Alix is completely abolished (Fig. 1a). These mice will be more fully described elsewhere. We first tested the effect of the absence of Alix on the downregulation of activated EGF Receptor (EGFR), which requires ESCRTs for sorting inside multivesicular bodies (MVBs) and is degraded in the lysosomes^33^. In wild type (wt) MEFs deprived of serum, addition of 100 ng/ml EGF led to an almost complete loss of EGFR within 6 h after EGF addition. As expected, this loss was inhibited by bafilomycin, a reagent known to block lysosome acidification and degradative activity (Fig. 1a). After 6 h, 23% of the receptor remained in Alix ko cells, compared to only 4.2% in wt cells. Theoretical fitted curves (dashed lines) were clearly different with a mean degradation rate of 0.78 for ko and 1.2 for wt cells, the overall EGFR degradation level being 32% lower in Alix ko cells. Noteworthy is that the difference in degradation rate is clearly seen in the first hour but disappears in the following hour (Fig. 1a,b).

A possible explanation for this early delay in EGFR degradation could be through perturbation of endocytosis or of endosomal function in the absence of Alix^14^,^34^. However, the distribution and morphology of the early endosomes, recycling endosomes, MVBs and late endosomes/lysosomes, as judged by analysis respectively of the early endosome antigen 1 (EEA1), Rab 11- or lysosomal associated membrane protein 1 (Lamp1)-positive compartments, appeared unaffected in Alix ko cells (Supplementary Fig. S1a). Depletion of Alix by siRNAs have been reported to decrease both LBPA levels and, as a consequence, cellular cholesterol levels, presumably because the storage capacity of endosomes is affected^35^. However we saw no difference in LBPA (Supplementary Fig. S1b) or filipin staining (Supplementary Fig. S1c) in Alix ko and wt MEFs showing that Alix ko cells do not suffer from major cholesterol defects.

**Figure 2:**
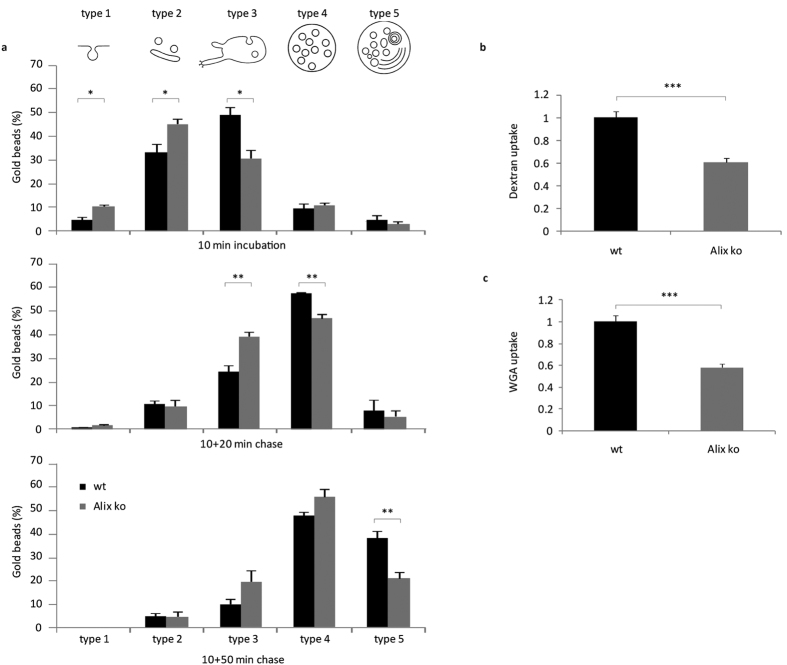
Endocytosis is delayed in Alix ko cells. (**a**) EM quantification of BSA-gold particle endosomal trafficking. MEFs were incubated with BSA-gold for 10 min at 37 °C and fixed immediately (top panel), or washed and further incubated at 37 °C for 20 min (middle panel) or 50 min (bottom panel). 1000 gold particles were counted for each condition and were attributed to each compartment type: type 1: plasma membrane invaginations, type 2: endocytic vesicles, type 3: early endosomal compartments, type 4: multivesicular bodies (MVB), type 5: late endosomes/ lysosomes (*P < 0.05; **P < 0.01, two-tailed Student’s t-test). (**b**) MEFs were incubated 10 min with dextran-TRITC at 37 °C, washed and fixed. Mean fluorescent values per cell were estimated using ImageJ (Number of cells: wt, n = 123; Alix ko, n = 116 in 3 independent experiments ***P < 0.001, two-tailed Mann–Whitney U test). (**c**) MEFs were stained for 5 min with fluorescent WGA at 4 °C, washed and incubated 5 min at 37 °C. Mean fluorescent values per cell were estimated using ImageJ (Number of cells: wt, n = 114; Alix ko, n = 107 in 3 independent experiments ***P < 0.001, two-tailed Mann–Whitney U test).

Using electron microscopy (Supplementary Fig. S2), we have been able to follow the uptake and endosomal trafficking of 6 nm BSA-gold particles in MEFs. Endocytic structures, labelled by bovine serum albumine (BSA)-gold were grouped into five categories according to the classification of Möbius *et al.*^36^, from type 1 structures corresponding to plasma membrane invaginations through to type 5 structures corresponding to lysosomes (see Fig. 2a). As shown in Supplementary Fig. S2, we did not detect any gross morphological abnormalities of endosomes and lysosomes in Alix ko cells. The relative numbers of type 3, 4 and 5 structures were equivalent between wt and Alix ko cells (Supplementary Fig. S2g) and the mean diameter of MVBs and lysosomes was unchanged (Supplementary Fig. S2h,i). The only noticeable difference between the two cell types was a small but significant decrease in the number of intraluminal vesicles (ILV) per MVB in Alix ko cells, with no detectable change in their size (Supplementary Fig. S2j,k).

### Loss of Alix affects fluid phase endocytosis but not trafficking through endosomes

To detect possible impairments due to the lack of Alix expression, wt and Alix ko MEFs were incubated with BSA-gold particles for 10 min and then washed and “chased” for a further 20 or 50 min (Fig. 2a). In wt cells, after 10 min incubation the majority of particles were already found in closed vesicles and early endosomes (type 2 and 3) (Fig. 2a, upper panel). After 20 min chase (Fig. 2a, middle panel), the particles were mainly detected in MVBs (type 4) and after 50 min (Fig. 2a, lower panel), a significant proportion was seen in late endosomes/lysosomes (type 5), reflecting the expected endosomal trafficking of the BSA-gold particles. In Alix ko cells, a significant delay in trafficking to early endosomes was seen as early as at 10 min with around 40% fewer particles found in this compartment (type 3), (Fig. 2a, upper panel). The delay in trafficking was maintained throughout the 20 and 50 min chase periods, as revealed by fewer particles in type 4 and type 5 compartments, respectively (Fig. 2a, middle and lower panel). Thus taken together these data show that absence of Alix has a mild effect on the early steps of fluid phase endocytosis without interfering with the subsequent steps along the late endosomal pathway.

We confirmed this effect on non-selective, constitutive endocytosis by showing that uptake of fluorescent dextran was reduced by 40% in Alix ko compared to wt cells (Fig. 2b). Furthermore, endocytosis of fluorescent wheat germ agglutinin (WGA), a lectin which binds to gangliosides and most cell surface glycoproteins, was also reduced by 45% in Alix ko cells (Fig. 2c). Thus, Alix deletion affects constitutive bulk flow endocytosis.

### Clathrin-independent endocytosis is selectively disrupted in Alix ko cells

**Figure 3:**
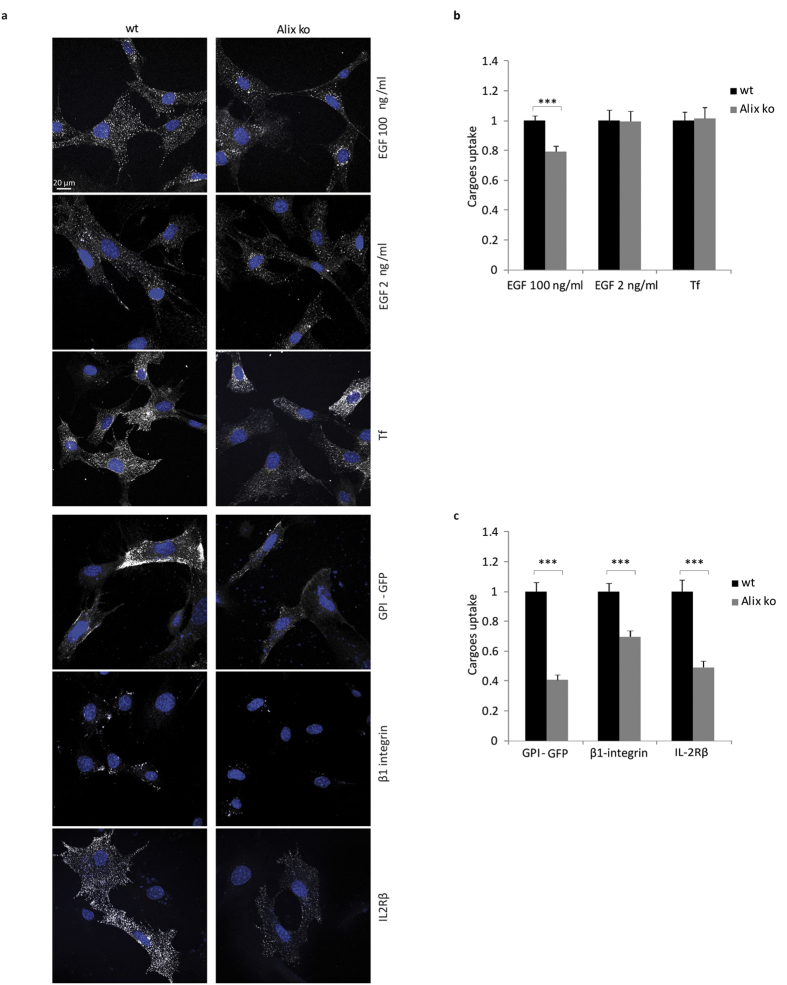
CIE is selectively disrupted in Alix ko cells while CME is unaffected. (**a**) Representative photographs of wt and Alix ko MEFs after endocytosis of EGF, Tf, GPI-GFP, β1 integrin and IL2Rβ. Serum-starved MEFs were incubated with EGF-TRITC (EGF, 2 or 100 ng/ml) or transferrin-TRITC (Tf) at 4 °C, followed by 10 min incubation at 37 °C. MEFs expressing GPI-GFP or IL2Rβ-GFP were incubated with anti-GFP antibodies at 4 °C, followed by 5 min incubation at 37 °C. To follow β1-integrin endocytosis, MEFs were incubated at 4 °C with anti-active-β1 integrin antibodies followed by 10 min incubation at 37 °C. In both latter cases, cell surface bound antibodies were removed by acid treatment. Hoechst stained nuclei appear in blue. (**b**,**c**) Mean fluorescence values per cell were estimated using ImageJ. Number of cells in 3 independent experiments: EGF 100 ng/ml, wt, n = 161; Alix ko, n = 128; two-tailed Student’s t test; EGF 2 ng/ml, wt, n = 95; Alix ko, n = 85; Tf, wt, n = 87; Alix ko, n = 96; GPI-GFP, wt, n = 59; Alix ko, n = 82, two-tailed Mann–Whitney U test; β1-integrin, wt, n = 81; Alix ko, n = 76; two-tailed Student’s t-test; IL2Rβ, wt, n = 100; Alix ko, n = 102; two-tailed Mann–Whitney U test; ***P < 0.001).

In view of the lack of gross endosomal abnormalities in Alix ko cells, or any obvious impairment in endosome trafficking, we reasoned that the observed delay in EGFR degradation in these cells might result from a defect in EGFR endocytosis. We found that this is indeed the case since internalization of EGFR revealed by fluorescent EGF, was significantly decreased in Alix ko MEFs stimulated with 100 ng/ml EGF. In contrast, no delay in endocytosis could be detected with 2 ng/ml EGF (Fig. 3a,b). Interestingly, it was previously shown that low concentrations of EGF trigger CME of the EGFR, whereas higher concentrations also stimulate internalization of a fraction of the receptor by CIE^37^,^38^. Thus, these results provided an initial indication that loss of Alix may preferentially affect receptor uptake through a CIE pathway. Consistent with this conclusion, we found that uptake of fluorescent transferrin (Tf) via the Tf receptor (TfR), which is entirely clathrin-dependent, was unaffected by loss of Alix (Fig. 3a,b) whereas internalization of the β chain of the IL2R, the first physiological cargo shown to be endocytosed by CIE^39^, was significantly attenuated (Fig. 3a,c). To pursue this further, we studied the fate of glycosylphosphatidylinositol (GPI)-anchored GFP, since GPI-anchored proteins are typically endocytosed by CIE pathways^40^. Using GPI-GFP expressing MEF cells, we found that antibody-induced internalization of GPI-GFP was reduced by 60% in Alix ko cells compared to wt cells, providing an additional indication that Alix deletion severely impairs CIE (Fig. 3a,c). Finally, we studied internalization of β1 chain of integrins, which is important for cell adhesion and spreading, and which occurs through both CME and CIE mechanisms^41^,^42^. Endocytosis of β1 integrins was followed using an antibody against the activated form of the protein and was found to be significantly impaired in Alix ko cells although to a lesser degree than was seen for IL2R and GPI-anchored GFP (Fig. 3a,c). Consistent with this delayed uptake of β1 integrin, we observed a 25% increase in cell spreading of Alix ko MEFs 2 h after seeding on fibronectin (Supplementary Fig. S3).

### Alix deletion affects CTxB CIE

**Figure 4:**
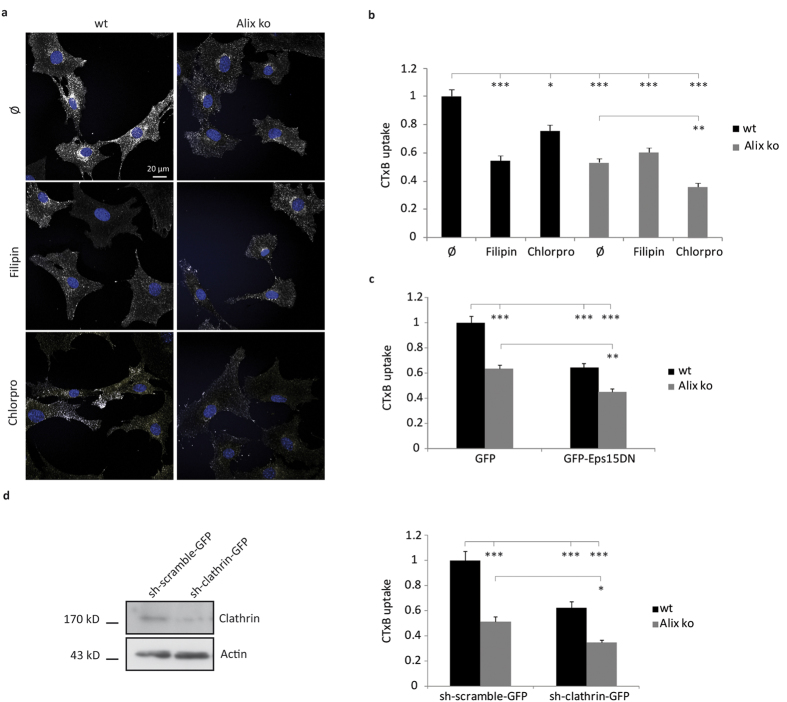
CIE but not CME of CTxB is impaired in Alix ko cells. (**a**) Representative images of 5 min CTxB-TRITC uptake by wt and Alix ko MEFs. The reduced endocytosis of CTxB-TRITC seen in Alix ko cells is further decreased by chlorpromazine (chlorpro) but not by filipin. Ø: no treatment. Hoechst stained nuclei appear in blue. (**b**) Quantification of the experiment shown in (**a**). Mean fluorescence values per cell were calculated using ImageJ (Number of cells in 3 independent experiments: wt Ø, n = 107; wt filipin, n = 90; wt chlorpro, n = 72; Alix ko Ø, n = 145; Alix ko filipin, n = 100; Alix ko chlorpro, n = 95; *P < 0.05; **P < 0.01; ***P < 0.001, Dunn’s multiple comparison test). (**c**) Quantification of 5 min CTxB uptake by wt and Alix ko MEFs expressing GFP or GFP-Eps15DN. Mean fluorescence values per cell were calculated using ImageJ (Number of cells in 5 independent experiments: wt GFP, n = 147; wt GFP-Eps15DN, n = 93; Alix ko GFP, n = 147; Alix ko Eps15DN, n = 105; **P < 0.01; ***P < 0.001, Dunn’s multiple comparison test). (**d**) Quantification of CTxB uptake by wt and Alix ko MEFs expressing sh-clathrin-GFP. Wt and Alix ko MEF cells were transfected with an sh vector directed against clathrin (sh-clathrin-GFP) or a control vector (sh-scramble-GFP). 72 h later, cells were incubated with CTxB for 20 min at 4 °C, followed by 5 min at 37 °C and internalized CTxB was quantified. (Number of cells in 4 independent experiments: wt sh-scramble-GFP, n = 62; wt sh-clathrin-GFP, n = 68; Alix ko sh-scramble-GFP, n = 60; Alix ko sh-clathrin-GFP, n = 63; *P < 0.05; ***P < 0.001, Dunn’s multiple comparison test). Western blot analyses using anti-clathrin antibody demonstrate the efficacy of the sh-clathrin plasmid in downregulating clathrin expression in N2a cells 48 h after transfection.

The CIE pathway is known to be hijacked by toxins such as CTx. The pentameric B chain of CTx (CTxB) binds to GM1 gangliosides at the plasma membrane leading to nucleation of lipid nanodomains, so-called “lipid rafts”, and endocytosis of the toxin^43^,^44^. Uptake of CTxB through CIE was confirmed in wt MEFs, since pretreatment of the cells with filipin, a reagent which sequesters membrane cholesterol thereby preferentially blocking CIE, reduced uptake of the toxin by around 50% (Fig. 4a,b). Consistent with the finding that to a lesser extent CTxB can also enter cells through the CME pathway^2^, chlorpromazine treatment, which blocks CME, reduced CTxB uptake by around 25% (Fig. 4a,b). CTxB internalization was similarly reduced in cells expressing a mutated form of epidermal growth factor receptor substrate 15 (Eps15DN) known to block CME^45^ or in cells depleted of clathrin (Fig. 4c,d). Interestingly, in untreated Alix ko cells, the amount of CTxB endocytosed was reduced by around 40% compared to wt cells, similar to the level observed in wt cells treated with filipin. Furthermore, treatment of Alix ko cells with filipin had no further inhibitory effect on CTxB uptake (Fig. 4a,b). In contrast, treatment of Alix ko cells with chlorpromazine, expression of Eps15DN, or clathrin depletion, led to reduction in CTxB endocytosis similar to that seen in wt cells (Fig. 4b–d). Control experiments showed that CTxB binding to the plasma membrane of wt and Alix ko cells was similar at 4 °C (Supplementary Fig. S4a). Furthermore, CTxB uptake was already affected after 1 min (Supplementary Fig. S4b) and GPI-GFP recycling was not changed (Supplementary Fig. S4c), making it unlikely that the decrease in CTxB endocytosis by Alix ko MEFs is due to accelerated recycling of the cargo. All these results, taken together with the normal endocytosis of TfR in Alix ko cells (Fig. 3a,b), demonstrate that loss of Alix principally affects the CIE pathways without impairing CME.

Following internalization, CTxB is known to traffic to the Golgi^44^. In both wt and Alix ko cells, after prolonged incubation the endocytosed CTxB co-localized with the Golgi marker GM130 (Supplementary Fig. S4d), showing that, even though delayed (Supplementary Fig. S4e), trafficking of the toxin down to the Golgi occurs in the absence of Alix.

### Both Alix and endophilin-A relocalize to membrane patches containing CTxB

**Figure 5:**
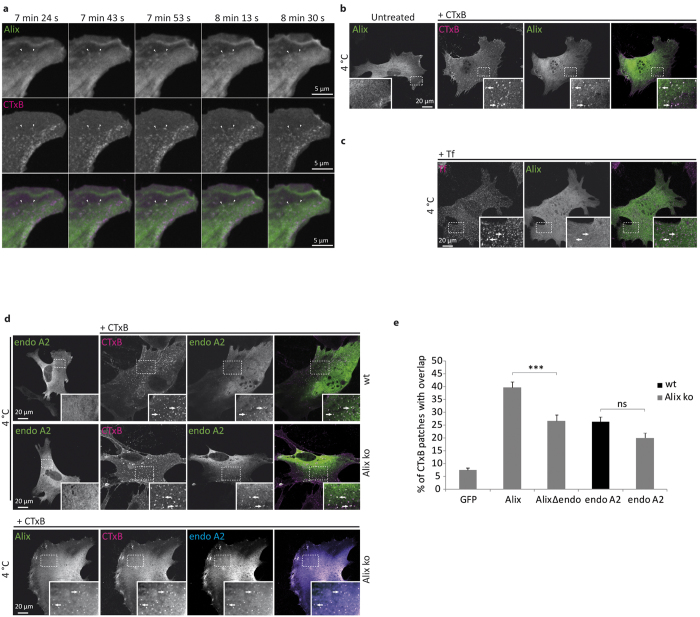
Alix and endophilin A2 relocalise to CTxB labeled membrane patches. (**a**) Images from time-lapse video microscopy, acquired by spinning-disk confocal microscopy of an Alix ko MEF expressing GFP-Alix 5 min after addition of CTxB-TRITC. Arrows indicate GFP-Alix (green) recruitment at the level of preexisting (right) or appearing (left) CTxB labeled patches (magenta). Both arrows show disappearance of CTxB and GFP-Alix. (**b**) Recruitment of Alix (green) to CTxB patches (magenta). Alix ko MEFs expressing GFP-Alix were left untreated, or incubated for 20 min with CTxB-TRITC at 4 °C prior to fixation. (**c**) Alix (green) is not recruited to membrane patches containing TfR (magenta). Alix ko MEFs expressing GFP-Alix were incubated for 20 min with Tf-TRITCat 4 °C prior to fixation. (**d**) Upper panels: Endophilin A2 recruitment to CTxB patches in both wt and Alix ko cells. wt MEFs and Alix ko MEFs expressing Myc-endophilin-A2 (endo A2), were left untreated, or incubated with CTxB-TRITC for 20 min at 4 °C before fixation and immunostaining with anti-Myc antibodies. Lower panel: Endophilin A2 (blue) and Alix (green) colocalize to CTxB patches (magenta). Alix ko MEFs expressing GFP-Alix and Myc-endophilin were incubated with CTxB-TRITC for 20 min at 4 °C before immunolabeling with anti-Myc antibodies. The image on the right shows the 3-color merge. Arrows indicate representative patches containing all 3 proteins. (**e**) Graph showing the percentage of CTxB labeled patches colocalizing with GFP, GFP-Alix, Flag-AlixΔendo or Myc-endophilin-A2 in wt (black) or Alix ko cells (gray). Quantification was performed using ImageJ (Number of cells in 3 independent experiments: GFP, n = 31; Alix, n = 53; AlixΔendo, n = 43; endoA2, n = 57 for wt and n = 56 for Alix ko. ***P < 0.001, one way ANOVA and Dunett’s test).

In Alix ko MEFs expressing GFP-Alix, the fluorescent signal was homogeneously distributed throughout the cytoplasm, albeit with somewhat higher local concentrations at certain regions of the plasma membrane particularly around the leading edge (Fig. 5b left panel). Live cell imaging of Alix ko cells expressing GFP-Alix, showed that Alix was transiently recruited to discrete spots at the plasma membrane (Supplementary video 1). No recruitment of GFP was seen in cells expressing GFP alone (Fig. S4f). In cells incubated with CTxB, Alix relocalization occurred more frequently and preferentially at CTxB positive patches (Fig. 5a). Interestingly GFP-Alix recruitment followed the appearance of CTxB labelled patches and terminated with the disappearance of CTxB, suggesting that it occurs at sites of CTxB entry (arrows in Fig. 5a and Supplementary Video2). In cells incubated with CTxB at 4 °C to block endocytosis, Alix was found on discrete, ~400 nm patches of the plasma membrane which in most cases were also decorated by the toxin (Fig. 5b). About 40% of the sites occupied by CTxB were also positive for GFP-Alix (Fig. 5e). Tf-TRITC (tetramethylrhodamine isothiocyanate) incubated on MEFs at 4 °C also concentrated in patches, which were smaller than CTxB positive patches and never labelled with GFP-Alix (Fig. 5c).

CTxB bound to GM1 gangliosides of the plasma membrane becomes associated with detergent resistant membrane fractions (DRMs)^46^. To investigate this further, Alix ko MEFs expressing GFP-Alix were exposed to CTxB-TRITC for 30 min at 4 °C, then incubated briefly with Triton-X100 to remove any cytosolic GFP-Alix. Analysis of the Triton-resistant fluorescence showed that most of the GFP-Alix had been lost, leaving behind GFP-Alix aggregates many of which were also labelled with CTxB (Supplementary Fig. S4g). In contrast, in the same cells exposed to transferrin (Tf-TRITC) which does not localize into DRMs, extraction with Triton-X100 completely eliminated the Tf-TRITC labelling as well as most of the GFP-Alix (Supplementary Fig. S4g). Thus, our results show that Alix is recruited to the detergent resistant lipid microdomains which are formed at the plasma membrane upon CTxB binding, and which probably correspond to the sites at which the toxin is internalized^4^.

We next tested whether relocalization of Alix to CTxB-induced membrane patches requires binding to endophilin-A, which has also been shown to be recruited to these sites^3^,^4^. As shown in Fig. 5d (upper panel), Myc-tagged endophilin-A2 was indeed recruited to membrane patches induced by CTxB. The recruitment of endophilin-A2 to CTxB-membrane patches occurred in both wt and Alix ko cells indicating that the endophilin-A2 relocalization is not dependent on the presence of Alix (Fig. 5d,e). Nevertheless, co-expression of Myc-endophilin-A2 and GFP-Alix in Alix ko cells showed that both proteins colocalized to the CTxB-induced patches (Fig. 5d lower panel).

Alix interacts with the SH3 domain of endophilin-A through a proline-rich domain (P^748^–P^761^)^12^. A mutant protein lacking this domain (AlixΔendo) was expressed in Alix ko cells and was also found to relocalize to CTxB patches, although less strongly than wt Alix (Fig. 5e). Thus endophilin-A and Alix are both recruited independently to plasma membrane patches formed upon CTxB binding, although the binding of Alix may be stabilized, or its recruitment to these sites facilitated by the presence of endophilin-A.

### Alix and endophilin-A co-operate in mediating CIE

**Figure 6:**
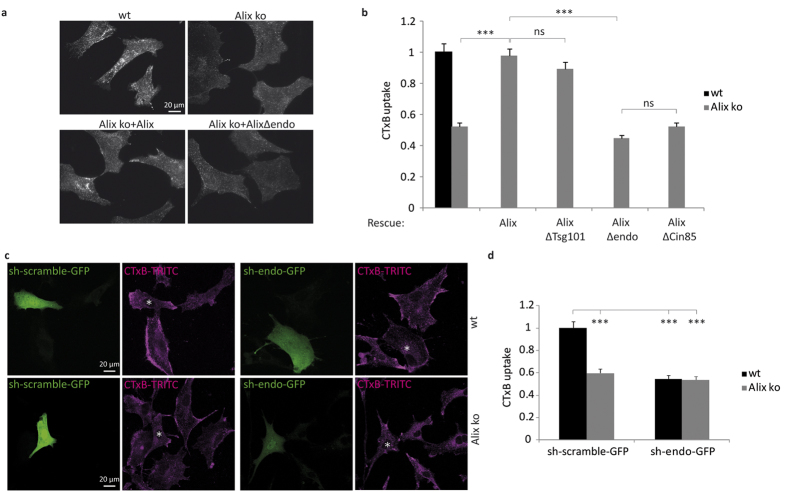
Alix and endophilin A2 mediate CIE of CTxB. (**a**) Alix mediated CIE of CTxB requires binding to endophilins. CTxB-TRITC uptake by wt and Alix ko MEFs (upper panel) or by Alix ko cells transduced with viruses (lower panel) encoding for Alix (Alix ko + Alix), or for a mutant of Alix unable to bind to endophilin-A (Alix ko + AlixΔendo). Cells were incubated with CTxB during 20 min at 4 °C, followed by 5 min at 37 °C and acid stripping. (**b**) Quantification of CTxB uptake using the same type of experiments as shown in (**a**). Uptake of CTxB was quantified in wt or Alix ko cells or in Alix ko cells transduced with Alix or Alix deletion mutants lacking several amino-acids of the PRD corresponding to binding sites for Tsg101 (AlixΔTsg101: AlixΔP^717^–P^720^), endophilin-A (AlixΔendo: AlixΔP^748^–P^761^), or Cin85 (AlixΔCin85: AlixΔP^739^–R^745^). Mean fluorescence values per cell were estimated using ImageJ. (Number of cells in 4 independent experiments: wt, n = 104; Alix ko, n = 98; rescue Alix, n = 112; AlixΔTsg101, n = 117; AlixΔendo, n = 147; AlixΔCin85, n = 133. ***P < 0.001, Dunn’s multiple comparison test). (**c**) Endophilin-A2 and Alix act in a common pathway in CIE of CTxB. wt and Alix ko MEFs were transfected with plasmids containing sh-endophilin and coding for GFP (sh-endo-GFP) or a scrambled version of the same shRNA sequence (sh-scramble-GFP), and cultured for 72 h. Cells were then incubated with CTxB-TRITC (magenta) for 20 min at 4 °C, followed by 5 min at 37 °C. Surface bound toxin was removed by acid treatment prior to fixation and processing. Asterisks indicate the nuclei of transfected cells. (**d**) Quantification of the experiments shown in (**c**). CTxB mean fluorescence values per transfected cell were calculated using ImageJ software (Number of cells in 4 independent experiments: wt sh-scramble-GFP, n = 93; wt sh-endo-GFP, n = 70; Alix ko sh-scramble-GFP, n = 76; Alix ko sh-endo-GFP, n = 67. ***P < 0.001, one way ANOVA and Dunnett’s test).

Rescue experiments using viral transduction (Supplementary Fig. S5a) showed that the impairment in CTxB CIE observed in Alix ko cells is due solely to the absence of Alix, since restoring Alix expression fully restored the capacity of Alix ko cells to internalize CTxB (Fig. 6a,b). In sharp contrast, expression of AlixΔendo was not able to rescue CTxB endocytosis by Alix ko MEFs. This result, together with the fact that deletion of the endophilin-SH3 binding site (P^748^–P^761^) does not impair binding to other Alix-interacting proteins^12^,^23^,^47^,^48^ demonstrates that interaction between Alix and endophilin-A is necessary in order to drive CIE of CTxB (Fig. 6a,b). Alix mutants deleted of other discrete regions of the PRD necessary for binding of Tsg101 (P^717^–P^720^) or CIN85 (P^739^–P^745^) were also tested for their capacity to rescue CTxB uptake by Alix ko cells. AlixΔTsg101 but not AlixΔCIN85 was able to rescue CTxB endocytosis (Fig. 6b) showing that binding of Alix to Tsg101 of ESCRT-I is not required for Alix to mediate CTxB CIE, whereas interactions with endophilins and CIN85, which have been both reported to mediate CIE^3^ are mandatory for Alix function in this process.

The endophilin-A2 isoform expressed in fibroblasts^49^ was downregulated using an sh-containing plasmid (sh-endo-GFP) (Supplementary Fig. S5c) and in accordance with the findings of Renard *et al.*^4^ and Boucrot *et al.*^3^, depletion of endophilin-A2 reduced CTxB endocytosis by more than 40% in wt MEFs (Fig. 6c,d). CTxB uptake was further reduced by chlorpromazine but not by filipin, suggesting that endophilin-A2 depletion blocked CIE, but not CME of CTxB (Supplementary Fig. S5d). In sharp contrast to wt cells, knocking-down endophilin-A2 in Alix ko cells did not reduce CTxB uptake any further (Fig. 6c,d), demonstrating that Alix and endophilin both act in the same pathway to mediate CIE. Noteworthy, is that the level of endophilin-A2 was equivalent between Alix ko and wt MEFs, showing that the effects observed in Alix ko cells do not simply result from a lack of endophilin expression due to the absence of Alix (Supplementary Fig. S5b).

**Figure 7:**
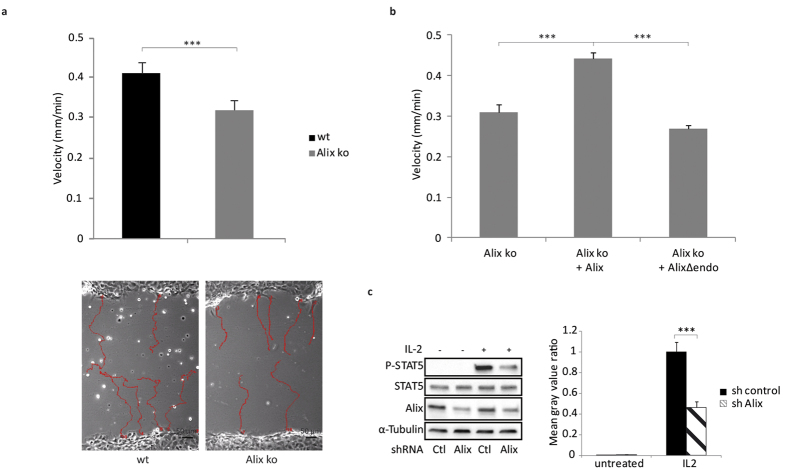
Physiological consequences of loss of Alix expression. (**a**) Decreased motility of Alix ko cells in a wound healing assay. Cells migrating into the gap were observed for 20 h and migration rates were measured using Metamorph (Number of cells in 2 independent experiments: wt and ko, n = 200; ***P < 0.001, two-tailed Student’s t-test). Representative recordings of migration tracks are shown in the lower panel. (**b**) Phenotypic rescue of Alix ko cells transduced with Alix (Alix ko + Alix) or AlixΔendophilin (Alix ko + AlixΔendo). Velocities were measured as in (**a**) (Number of cells in 2 independent experiments: Alix ko + Alix and Alix ko + AlixΔendo, n = 200; ***P < 0.001, one-way ANOVA and Tukey HSD). (**c**) Alix knock-down in EBV-B cells decreases IL2-induced phosphorylation of STAT5. Cells were transfected with pSuper-empty or pSuper-shAlix and stimulated with IL2 for 10 min. Immunoblots were performed to analyze the effect on tyrosine phosphorylation of STAT5. Band intensities were estimated using ImageJ. P-Stat5 and Stat5 intensities were normalized to α-tubulin intensities. Graph shows the ratio between P-Stat5 and Stat5 values (n = 3; ***P < 0.001, two-tailed Student’s t-test).

CIE has been shown to be required for cell migration^50^ and this has recently been confirmed in endophilin-A knock-down experiments^3^. We therefore used a wound healing assay to investigate the behavior of the Alix ko MEFs. As shown in Fig. 7a, the migration of the Alix ko MEFs was significantly reduced compared to wt cells, while restoration of Alix expression by viral transduction increased the velocity of Alix ko MEFs to the level seen in wt cells (Fig. 7b). In contrast, no increase in migration activity was seen when cells were transduced with AlixΔendo. The defect in migration caused by the absence of Alix therefore correlates with the ability of Alix to mediate endophilin-dependent CIE.

Given our finding that Alix is required for endocytosis of the IL2R β-chain (Fig. 3a,c), we decided to test whether Alix depletion also interferes with IL2R signaling. Binding of IL2 to its receptor activates a variety of downstream events including activation of JAK tyrosine kinases and subsequent phosphorylation of the transcription factor Signal transducer and activator of transcription 5 (STAT5). We found that knocking down Alix expression in the human Epstein-Barr virus B cell line (EBV-B), severely reduced IL2-induced STAT5 phosphorylation (Fig. 7c) consistent with a requirement of CIE of the activated IL2 receptor for the downstream signalling pathway leading to JAK/STAT5 activation.

## Discussion

Much of the machinery required for endocytic internalization of molecules at the plasma membrane relies on the activity of clathrin and its associated proteins in a process known as clathrin-mediated endocytosis (CME). However, fluid phase endocytosis, internalization of certain surface receptors as well as entry of some pathogenic toxins occur through mechanisms which do not require clathrin and have been collectively designated as clathrin-independent endocyotosis (CIE). The lack of known elements common to the cell entry processes has led to the view that distinct CIE pathways exist, although an understanding of the general mechanisms involved remain to be elucidated and blurs a general understanding of CIE. In this study, we have explored the role of Alix (ALG-2-interacting protein-X) in CIE. We have used MEFs prepared from the Alix homozygous knockout (Alix ko) mice recently obtained in our laboratory, and we demonstrate that Alix plays a central role in driving fluid phase endocytosis, entry of cholera toxin and endocytosis of surface receptors known to be internalized through CIE. On the other hand, uptake of transferrin via its receptor, which depends solely on CME, is unaffected by the absence of Alix. Endophilin-A has recently been reported to be required for internalization of cell-surface receptors such as those for EGF and IL2^3^ and for entry of bacterial toxins^4^. We now show that Alix acts in association with endophilin-A and CIN85 but not Tsg101 of ESCRT-I to promote CTxB endocytosis and that Alix ko cells are defective in cell migration, in accordance with a role of CIE in this process. Finally we present evidence that signaling through the IL2R is strongly impaired by Alix down-regulation, thus establishing for the first time a link between the internalization of IL2R and the activation of its downstream ligand-induced signaling function.

Based on its homology with the yeast protein Bro-1, which binds the ESCRT-III complex and is required for cargo sorting in MVBs, Alix has been suggested to be a major actor for MVB genesis in mammalian cells^14^. However, Doyotte *et al.* have already challenged this idea by showing that Alix down regulation has almost no effect on endosome morphology and trafficking^51^ in contrast to HD-PTP (His domain phosphotyrosine phosphatase), another Bro1/Alix related protein, which they found to be required for MVB formation. Several publications have reported that Alix down regulation increases^52^, has no effect^34^,^53^ or only delays EGFR degradation^54^,^55^, reinforcing the idea that Alix might regulate but is not necessary for making MVBs. In line with this, our present work show that Alix is not the mammalian ortholog of Bro-1 since, even though delayed, EGFR degradation occurs in Alix ko cells and since no major abnormalities can be seen in endosomes of these cells. The only abnormal feature of Alix ko endosomes is a slight decrease in the number of ILVs in MVBs, compatible with a possible regulatory role of Alix in ILV budding^14^ or backfusion^56^,^57^. However, this decrease could also be indirectly due to a modification of growth factor receptor activity, which in the case of EGFR, is known to regulate the number of ILVs within MVBs^58^. Delayed degradation of EGFR in Alix ko cells could result from the compromised EGFR endocytosis that we detected in the same cells. This impairment only occurred upon exposure to high concentrations of EGF (100 ng/ml), a condition known to favor CIE of the receptor^37^,^38^. In the presence of 2 ng/ml EGF, a concentration known to activate CME exclusively, no decrease in receptor internalization was seen in Alix ko compared to wt MEFs. Interestingly, Alix directly interacts with both endophilins and CIN85, which are known to form a complex regulating EGFR endocytosis at high EGF concentrations^32^. Alix was already reported to modulate the interaction between Cbl and CIN85 and thereby ubiquitination of the EGFR by Cbl^52^, which is known to regulate entry of the receptor via CIE^37^. More recently, Boucrot *et al.*^3^ reported a requirement not only for endophilins but also for CIN85 in CIE of the EGFR. These observations are therefore consistent with the hypothesis that Alix orchestrates a “ménage à trois” together with CIN85 and endophilins to promote EGFR uptake via CIE. This protein complex might also be involved in CIE of other cargoes as demonstrated by our finding that CTxB CIE is highly dependent on the capacity of Alix to bind the SH3 domain of endophilin-A and of CIN85. shRNA-mediated knock-down of endophilin-A did not further reduce endocytosis of CTxB in Alix ko cells, indicating that endophilins and Alix operate in the same pathway. While both proteins were recruited independently to sites of CTxB uptake, the interaction of Alix with endophilin-A seems to favor the recruitment or stabilize the presence of the latter to these sites. Endophilin-A binding to clathrin-independent endocytic sites can occur through their BAR domains^4^, which recognize concave cytoplasmic surfaces of the plasma membrane^6^, thus leaving SH3 domains free to interact with Alix. Possible mechanisms for recruitment of Alix to the plasma membrane may be through binding to ALG-2, a calcium-dependent interaction recently shown to mediate Alix recruitment following membrane damage^27^. Another possible mechanism is through binding to LBPA, or more probably to a related phospholipid, since to date there is no evidence that LPBA itself is present in the plasma membrane. Following binding to LBPA in endosomal membranes, Alix changes its conformation leading to the insertion of a hydrophobic loop into the cytoplasmic leaflet of the bilayer^15^. Thus, Alix insertion into the plasma membrane could potentially contribute to generation of membrane curvature and thereby facilitate the recruitment of endophilin-A through its BAR domain. However, we favor the hypothesis that its role is to organize other proteins which then deform membranes. Until today, Alix involvement in membrane deformation has been restricted to its capacity to recruit CHMP4B, which polymerizes into helices deforming the plasma membrane towards the outside to allow membrane repair, virus budding or cytokinesis^59^. During endocytosis, Alix may drive the assembly of the CIN85/endophilin complex at the plasma membrane required for its deformation and scission with the opposite topology to that induced by ESCRT-III complexes. Boucrot *et al.*^3^ have observed that endophilin-A controls only the internalization of ligand-activated receptors, such as EGFR and IL2R, but not that of GPI–anchored receptors or fluid phase endocytosis, which we have shown to be also dependent on Alix. Further work will be required to define whether endophilin binding to Alix is also mandatory for these endocytic processes.

The variety of cargoes which are internalized by Alix-driven CIE suggests a role for non-clathrin-mediated pathways in many cellular functions. We have established a correlation between the Alix-endophilin pathway and cell migration, consistent with the previously described role of CIE in this process^50^. Internalization of cell surface activated receptors is a widespread mechanism for regulating cell signaling, which can occur both at the cell surface or at the endosomal membrane. However currently, it is unclear how CIE or CME of surface-activated receptors affects the propagation of intracellular signals. In our case, Alix knockdown strongly reduced STAT5 phosphorylation. The only endocytic route of IL2R is through CIE^39^ and the absence of Alix severely impaired endocytosis of the β-chain of the receptor. Thus, our results are consistent with CIE being necessary for IL2R signaling.

The dual role of endophilins in both CIE and CME is no doubt partly responsible for previous conflicting results, and blurs our present understanding of these pathways in normal physiology, in particular in regulating cell signaling and synaptic vesicle formation, in which both processes are implicated^60^,^61^. The lethal phenotype observed in endophilin ko mice prevents use of the endophilin deletion model for resolving this issue. On the other hand, our finding that Alix mediates only CIE, without affecting CME, highlights the usefulness of Alix ko mice as a valuable and viable model with which to explore and understand more precisely the physiological role of CIE *in vivo.*

## Materials and Methods

### Antibodies and other reagents

Antibodies used were: monoclonal and polyclonal anti-FLAG antibodies from Sigma-Aldrich, polyclonal anti-Alix from Covalab, monoclonal anti-actin from Millipore, polyclonal antibodies against EGFR, Myc and EEA1 were from Santa Cruz, anti-GFP and anti-LAMP1 from Abcam, monoclonal anti-Rab11, monoclonal anti-clathrin and anti-GM130 from BD transduction, monoclonal anti-β1 (9EG7) was from BD Pharmingen, anti-STAT5 and anti- phosphorylated STAT5 were from Cell Signaling. Monoclonal anti-LBPA (6C4) and polyclonal anti-endophilin A antibodies were generous gifts from Jean Gruenberg (Geneva, Switzerland) and Pietro De Camilli (New Haven, United States) respectively. Monoclonal anti-IL2Rβ antibody (mAb 341) was described in^39^. Secondary antibodies conjugated to Alexa Fluor 488, Alexa Fluor 594 and Alexa 594-WGA, as well as EGF-TRITC, CTxB-TRITC, Tf-TRITC and Dextran-TRITC were from Molecular Probes. Horseradish peroxidase-conjugated goat anti-mouse and anti-rabbit secondary antibodies were from Jackson Laboratories. Filipin, chlorpromazine, EGF, bafilomycin A1 were from Sigma-Aldrich. 6 nm BSA-Gold tracer was from Aurion (The Netherlands).

### DNA constructs

Flag-Alix, Flag-AlixΔP^748^–P^761^ (AlixΔendo), Flag-AlixΔP^717^–P^720^ (AlixΔTsg101), and Myc-endophilin A2 cDNAs cloned in PCI (Promega) were described previously^12^,^20^. Flag-Alix ΔP^739^–R^745^ (AlixΔCIN85) was obtained by mutagenesis (Quick change II XL mutagenesis kit, Stratagene) using wt Alix as template and the following oligos:

sens: 5′-GGCATGCAGCAGCGACCATGCCGCCTGCTAAGC-3′

antisens: 5′-GCTTAGCAGGCGGCATGGTCGCTGCTGCATGCC-3′.

GFP-Flag-Alix was obtained by subcloning Flag-Alix cDNA into a pEGFP-C vector (Clontech). The GFP-GPI construct was a generous gift from G. Van der Goot (Lausanne, Switzerland). IL2Rβ-GFP was described in^39^. GFP-Eps15DN (GFP-EΔ95/295) described in^45^ was a kind gift of A. Benmerah (Paris, France). The small hairpin RNA (shRNA) expression vectors were constructed as follows: two complementary oligonucleotides were synthesised, annealed, ligated and inserted downstream of the human H1 promoter in the pSuperGFP expression vector (Oligoengine) using the BglII and HindIII restriction sites. For shRNA against mouse endophilin A2, the top strand sequence was 5′-GATCCCCGCCTTGACTTTGACTACAAGATTCAAGAGATCTTGTAGTCAAAGTCAAGGCTTTTTA-3′, and the bottom strand sequence was 5′-AGCTTAAAAAGCCTTGACTTTGACTACAAGATCTCTTGAATCTTGTAGTCAAAGTCAAGGCGGG-3′. As a negative control, a scrambled shRNA sequence was cloned into the same vector; the top strand sequence was 5′-GATCCCCATATACGTCACTTAGCGGCATTTCAAGAGAATGCCGCTAAGTGACGTATATTTTTTA-3′, and the bottom strand sequence was 5′-AGCTTAAAAAATATACGTCACTTAGCGGCATTCTCTTGAAATGCCGCTAAGTGACGTATA TGGG-3′.

For the Alix shRNA the top strand sequence was 5′-GATCCCCGCCGCTGGTGAAGTTCATCTTCAAGAGAGATGAACTTCACCAGCGGCTTTTTGGAAA-3′ and the bottom strand sequence was 5′-AGCTTTTCCAAAAAGTTCATCCAGCAGACTTACTCTCTTGAAGTAAGTCTGCTGGATGAACGGG-3′. For the clathrin (CHC1) shRNA, the top strand sequence was 5′-GATCCCCAACATTGGCTTCAGTACCTTGTTCAAGAGACAAGGTACTGAAGCCAATGTTTTTTTA-3′, and the bottom strand sequence was 5′-AGCTTAAAAAAACATTGGCTTCAGTACCTTGTCTCTTGAACAAGGTACTGAAGCCAATGTTGGG-3′.

All oligonucleotides were obtained from Invitrogen and all final expression constructs were confirmed by DNA sequencing.

### Generation of Alix ko fibroblasts

All experiments were performed in accordance with the relevant guidelines and regulation that were approved by the Committee on Animal Care of Institut des Neurosciences de Grenoble (GIN) and French legislation.

Alix ko mice were obtained in collaboration with the Clinique de la souris (Strasbourg, France). Briefly, exon 2 of *alix* (chromosome 9) was targeted by LoxP sites. Complete ko mice were obtained by backcrossing the floxed mice with CMV-Cre mouse. The recombination changes the reading frame introducing a stop codon at position 71 of the protein. Mouse embryonic fibroblasts (MEFs) were obtained as follows: E12.5 embryos were decapitated and eviscerated, trypsinized in 1 ml 0.25% trypsin, 0.53 mM EDTA (LifeTechnologies, Inc.) for 20 min at 37 °C and thoroughly minced. Cells were cultured in DMEM (Dulbecco’s modified Eagle’s medium) supplemented with 10% fetal bovine serum (FBS), 2 mM glutamine (Invitrogen). After mechanical dissociation, cells were plated in a 10-cm culture dish and cultured at 37 °C, in 5% CO_2_. MEFs from 3 individual embryos (wt or Alix ko) were pooled and frozen in liquid nitrogen.

### Rescue experiments

For rescue experiments, cDNAs were cloned in pBABE hygro (Addgene) and delivered via retroviral transduction following packaging in Phoenix-Eco cells, as recommended by their provider (ATCC, Manassas, VA). Supernatants containing viral particles from these cells were harvested, filtered through 0.45 μm filters, supplemented with 8 μg/ml polybrene (Sigma, St Louis, MO) and then used to transduce fibroblasts.

### Cell culture, transfections and treatments

MEF and N2a cells were cultured in DMEM, supplemented with 10% FBS and 2 mM glutamine. For transfections, 4.10^4^ cells were plated in 35 mm dishes and transfected 24 h later using Jet PEI PolyPlus (InvitroGene, France). DNA transfections were analysed after 24 h; shRNA transfections were analysed after 72 h. N2a cells were used to test the efficacy of sh endophilin and sh clathrin in down-regulating expression of endogenous endophilin-A2 and clathrin respectively.

EBV-B cells, immortalized from control patients as described in^62^, were a kind gift of J.L. Casanova (Paris). The cells were maintained in RPMI 1640 medium (Gibco) supplemented with 10% bovine fetal serum.

EBV-B cells were transfected with the plasmids indicated using the NeonTM transfection system (Invitrogen). 72 h after transfection, cells were either left untreated or treated with 1000 U/ml of human IL2 (kind gift of Dr Vogt) at 37 °C for 10 minutes. For biochemical analyses, cells were washed with ice cold PBS and lysed in SDS Sample Buffer 1X. Total lysates were analyzed by SDS-PAGE and Western blot analysis. To detect pSTAT5 activation in EBV-B cells, immunoblot signals were enhanced by Supersignal West Dura (Pierce).

### Immunofluorescence

MEFs were seeded at 4.10^4^ cells per 35 mm dish and grown on coverslips at 37 °C. Cells were washed twice with PBS and fixed by 4% paraformaldehyde (PFA) in PBS for 15 min. After permeabilisation with 0.25% Tween in PBS containing 3% BSA for 30 min, antibodies were applied for 1 h at room temperature, then developed using goat anti-mouse or goat anti-rabbit immunoglobulin G coupled to Alexa 594 or Alexa 488, diluted in PBS/0.25%Tween. Nuclei were stained using Hoechst 33258 (Sigma). Coverslips were mounted in Mowiol (Calbiochem) and cells were examined by confocal microscopy (see below).

### Internalization assays

For internalization experiments, MEFs were seeded on coverslips at 4.10^4^ cells per 35 mm dish.

#### CTxB internalization

MEFs were washed with cold DMEM containing 0.2% BSA and 20 mM Hepes, and incubated with 2 μg/ml CTxB-TRITC for 20 min at 4 °C. Unbound proteins were removed by washing with cold PBS containing 0.2% BSA and cells were then incubated for different times at 37 °C in DMEM containing 0.2% BSA to allow internalization. After washing with cold PBS containing 0.2% BSA, cells were fixed for 15 min with 4% PFA, washed with PBS and processed for microscopic examination. Where appropriate, surface bound ligands were removed by treatment with 25 mM sodium acetate in DMEM pH 2 for 2 min. The medium was then neutralized by addition of 25 mM Tris in DMEM, pH 10 and processed as described above. To study CTxB uptake in the presence of filipin (1 μg/ml) and chlorpromazine (10 μg/ml), cells were pre-treated with reagent for 30 min at 37 °C followed by 20 min at 4 °C in DMEM containing 0.2% BSA and 20 mM HEPES, as well as during the CTxB uptake period at 37 °C. The samples were imaged using a Zeiss axiovert 200 microscope equipped with a 20× or 40× objective or by confocal microscopy (see below) using 63× or 40× oil immersion objectives.

For uptake quantification, images were background subtracted, cellular regions of interest were marked, and the mean fluorescence intensity was measured in these regions using ImageJ. Intensities were normalized to binding of CTxB at 4 °C. For each condition, results are expressed as a fraction of uptake by wt cells, considered as 1.

#### EGF-TRITC, transferrin-TRITC, GPI-GFP, IL2Rβ and β1 integrin internalization

MEFs were rinsed with cold DMEM containing 0.2% BSA and 20 mM HEPES and incubated for 45 min on ice with EGF-TRITC (100 ng/ml or 2 ng/ml), monoclonal anti-GFP (4 μg/ml), monoclonal anti-IL2Rβ, monoclonal anti-β1 integrin antibody (4 μg/ml), or for 20 min on ice with 40 μg/ml transferrin-TRITC. Cells were then incubated at 37 °C for the indicated times to allow endocytosis. For EGF-TRITC and transferrin-TRITC, MEFs were starved in medium lacking serum for 4 h before incubation at 4 °C with ligands. For GPI-GFP, IL2Rβ-GFP and β1 integrin, internalized antibody was detected using a fluorescently labelled secondary antibody. Internalization, stripping, fixation and permeabilization were performed as described for CTxB.

#### Dextran-TRITC internalization

cells were incubated for 10 min at 37 °C with 3 mg/ml Dextran in internalization medium (5 mM glucose), washed 5 times with PBS containing BSA and fixed with 4% PFA. Internalized dextran was quantified using ImageJ.

#### WGA internalization

cells were washed with cold PBS containing 0.2% BSA and incubated 5 min at 4 °C with 2 μg/ml WGA-A594. Internalization was quantified as for CTxB.

For quantifications of GPI-GFP, IL2R-GFP, WGA, CTxB and β1 integrin endocytosis, the fluorescence detected inside cells after internalization at 37 °C was normalized to the fluorescence detected at 4 °C.

### Fluorescent microscopy

Images were acquired using a Zeiss Axiovert microscope controlled by Metamorph, using 20× or 40× objectives (for uptake quantification), or with a Zeiss LSM 710 inverted confocal microscope controlled Zen software, using a 40× oil objective (NA 1.3, Zeiss) or a 63× oil objective (NA 1.4, Zeiss), or 100× oil objective (NA 1,4, Zeiss). For endophilin/Alix colocalization (Fig. 5d), a Zeiss LSM 880 inverted confocal Airyscan microscope with a 63× oil objective (NA 1.4, Zeiss) was used. Laser lines at 488 nm, 561 nm and 633 nm were used for exciting fluorophores. Pinhole size was usually set to generate 1 μm-thick optical slices at all wavelengths.

### Internalization of BSA-gold

The BSA-gold uptake studies were performed according to^63^. Briefly, MEFs were incubated with 6 nm BSA-gold particles for 10 min at 37 °C, washed extensively with cold medium and then either fixed immediately, or incubated further at 37 °C for 20 min or 50 min before fixation. Cells were fixed overnight at 4 °C in 0.1 M phosphate buffer (PB) containing 2% PFA and 0.2% glutaraldehyde. After fixation, cells were detached by careful scraping and collected by centrifugation. The cell pellets were washed with PB, embedded in 10% (wt/vol) gelatin and infused with 2.3 M sucrose^64^. Gelatin blocks were frozen in liquid nitrogen and ultrathin cryo sections were cut at −120 °C using an Ultracut FCS ultracryomicrotome (Leica-Reichert). Sections were retrieved in a 1:1 solution of 2.3 M sucrose and 2% methyl cellulose. The sections were viewed at 80 kV in a JEOL 1200 EX electron microscope and imaged using an Olympus (Veleta, SIS) camera.

To follow vesicle trafficking, at each time point 40 cell profiles of 3 different grids were randomly selected, and gold particles present in the endocytic structures were assigned to compartments of types 1–5 as described in the Results section. For each time point, approximately 1000 beads were counted and BSA-gold counts in each compartment were expressed as a percentage of the total number of gold beads counted in the endocytic pathway.

### Colocalisation analysis

Images of CTxB patches were acquired and superimposed with the distribution of selected proteins. Images of regions of interest were thresholded to see only the patches of CTxB and the same threshold was then applied for all proteins. Mander’s coefficient was calculated using the JACOB plug-in of the ImageJ software^65^.

### EGFR degradation assay

MEFs were serum-starved for 16 h in DMEM containing 2% glutamine and incubated with 100 ng/ml EGF for 30 min, 1 h, 2 h, 4 h or 6 h. For bafilomycin treatment, cells were pretreated with 100 nM bafilomycin 2 h before and during incubation with EGF. Protein bands were quantified using the ImageJ plot profile tool. Degradation curves were fitted with the single exponential function: y = (1 − a)e^(−b*x)^ + a, where a = non degradable fraction, b = rate of degradation, x = time.

### Western Blotting

Cells were lysed in radioimmune precipitation assay (RIPA) buffer containing protease inhibitors (Complete, Roche Molecular). The cell lysates were cleared by centrifugation at 14,000 *g* for 20 min, and proteins (20 μg of total protein per lane, or 40 μg for the EGFR degradation assay) were separated by 10% SDS-PAGE and transferred to a polyvinylidene difluoride (PVDF) membrane (Millipore). The membranes were blocked with 5% milk in TBS (Tris buffered saline) containing 0.1% Tween and incubated with primary antibodies overnight at 4 °C, followed by incubation with secondary HRP-conjugated antibodies for 1 h at room temperature. Immunoreactive bands were revealed using the ECL detection reagent. Protein bands were quantified using the ImageJ plot profile tool.

### Wound healing assay

MEFs were seeded at 5.10^5^ cells per 35 mm dish. Confluent monolayers were scratched using a plastic pipette tip to simulate the wound. Cells were then washed with PBS, and cultured in serum-containing medium at 37 °C to allow wound healing. Images were recorded immediately after scratching and at 15 min intervals for 48 h with a Zeiss Axiovert 200 microscope using the 10× objective. For each cell type, ten cells in each of 10 fields were tracked using Metamorph and the velocity of each cell was calculated. Data were compiled from two independent experiments.

### Statistical Analyses

Results shown are means, and error bars represent standard errors of the mean (s.e.m). Except where otherwise stated, experiments were repeated at least 3 times. Statistical analyses were performed using Microsoft Excel and Prism 6 (GraphPad Software). The Gaussian distribution of the data were tested using Kolmogorov–Smirnov test (with Dallal–Wilkinson–Lillie for P value). In case of non-Gaussian distribution, the following non-parametric tests were used: two-tailed Mann–Whitney U test to compare two conditions, or one-way ANOVA (Kruskal–Wallis test) with a Dunn’s test to compare more than two data groups. In case of Gaussian distribution, two-tailed Student’s t-test was used for the comparison of the means in case of comparison between two conditions and parametric one-way ANOVA with a Tukey HSD or Dunnett’s multiple comparison tests in case of comparisons between more than two data groups. Significance of mean comparison is represented on the graphs by asterisks. All error bars denote s.e.m.

## Additional Information

**How to cite this article**: Mercier, V. *et al.* ALG-2 interacting protein-X (Alix) is essential for clathrin-independent endocytosis and signaling. *Sci. Rep.* **6**, 26986; doi: 10.1038/srep26986 (2016).

## Supplementary Material

Supplementary Video 1

Supplementary Video 2

Supplementary Information

## References

[b1] Doherty G. J. & McMahon H. T. Mechanisms of endocytosis. Annu Rev Biochem 78, 857–902, 10.1146/annurev.biochem.78.081307.110540 (2009). 19317650

[b2] Mayor S., Parton R. G. & Donaldson J. G. Clathrin-independent pathways of endocytosis. Cold Spring Harbor perspectives in biology 6, 10.1101/cshperspect.a016758 (2014). PMC4031960 24890511

[b3] Boucrot E. *et al.* Endophilin marks and controls a clathrin-independent endocytic pathway. Nature, 10.1038/nature14067 (2014). 25517094

[b4] Renard H. *et al.* Endophilin-A2 functions in membrane scission in clathrin-independent endocytosis. Nature, 10.1038/nature14064 (2014). PMC4342003 25517096

[b5] Ringstad N., Nemoto Y. & De Camilli P. The SH3p4/Sh3p8/SH3p13 protein family: binding partners for synaptojanin and dynamin via a Grb2-like Src homology 3 domain. Proc Natl Acad Sci USA 94, 8569–8574, 10.1073/pnas.94.16.8569 (1997). 9238017 PMC23017

[b6] Peter B. J. *et al.* BAR domains as sensors of membrane curvature: the amphiphysin BAR structure. Science 303, 495–499, 10.1126/science.1092586 (2004). 14645856

[b7] Gad H. *et al.* Fission and uncoating of synaptic clathrin-coated vesicles are perturbed by disruption of interactions with the SH3 domain of endophilin. Neuron 27, 301–312, 10.1016/S0896-6273(00)00038-6 (2000). 10985350

[b8] Milosevic I. *et al.* Recruitment of endophilin to clathrin-coated pit necks is required for efficient vesicle uncoating after fission. Neuron 72, 587–601, 10.1016/j.neuron.2011.08.029 (2011). 22099461 PMC3258500

[b9] Verstreken P. *et al.* Endophilin mutations block clathrin-mediated endocytosis but not neurotransmitter release. Cell 109, 101–112, 10.1016/S0092-8674(02)00688-8 (2002). 11955450

[b10] Llobet A. *et al.* Endophilin drives the fast mode of vesicle retrieval in a ribbon synapse. J Neurosci 31, 8512–8519, 10.1523/JNEUROSCI.6223-09.2011 (2011). 21653855 PMC3926091

[b11] Missotten M., Nichols A., Rieger K. & Sadoul R. Alix, a novel mouse protein undergoing calcium-dependent interaction with the apoptosis-linked-gene 2 (ALG-2) protein. Cell Death Differ 6, 124–129, 10.1038/sj.cdd.4400456 (1999). 10200558

[b12] Chatellard-Causse C. *et al.* Alix (ALG-2-interacting protein X), a protein involved in apoptosis, binds to endophilins and induces cytoplasmic vacuolization. J Biol Chem 277, 29108–29115, 10.1074/jbc.M204019200 (2002). 12034747

[b13] Kim J. *et al.* Structural basis for endosomal targeting by the Bro1 domain. Dev Cell 8, 937–947, 10.1016/j.devcel.2005.04.001 (2005). 15935782 PMC2862258

[b14] Matsuo H. *et al.* Role of LBPA and Alix in multivesicular liposome formation and endosome organization. Science 303, 531–534, 10.1126/science.1092425 (2004). 14739459

[b15] Bissig C. *et al.* Viral infection controlled by a calcium-dependent lipid-binding module in ALIX. Dev Cell 25, 364–373, 10.1016/j.devcel.2013.04.003 (2013). 23664863 PMC4129370

[b16] McCullough J., Fisher R. D., Whitby F. G., Sundquist W. I. & Hill C. P. ALIX-CHMP4 interactions in the human ESCRT pathway. Proc Natl Acad Sci USA 105, 7687–7691, 10.1073/pnas.0801567105 (2008). 18511562 PMC2409388

[b17] Sadoul R. Do Alix and ALG-2 really control endosomes for better or for worse. Bio. Cell 98 69–77, 10.1042/BC20050007 (2006). 16354163

[b18] Pires R. *et al.* A crescent-shaped ALIX dimer targets ESCRT-III CHMP4 filaments. Structure 17, 843–856, 10.1016/j.str.2009.04.007 (2009). 19523902 PMC2699623

[b19] Trioulier Y. *et al.* Alix, a protein regulating endosomal trafficking, is involved in neuronal death. J Biol Chem 279, 2046–2052, 101074/jbc.M309243200 (2004). 14585841 10.1074/jbc.M309243200

[b20] Mahul-Mellier A. L., Hemming F. J., Blot B., Fraboulet S. & Sadoul R. Alix, making a link between apoptosis-linked gene-2, the endosomal sorting complexes required for transport, and neuronal death *in vivo*. J Neurosci 26, 542–549, 10.1523/JNEUROSCI.3069-05.2006 (2006). 16407552 PMC6674414

[b21] Mahul-Mellier A. L. *et al.* Alix and ALG-2 are involved in tumor necrosis factor receptor 1-induced cell death. J Biol Chem 283, 34954–34965, 10.1074/jbc.M803140200 (2008). 18936101 PMC3259881

[b22] Strack B., Calistri A., Craig S., Popova E. & Gottlinger H. G. AIP1/ALIX is a binding partner for HIV-1 p6 and EIAV p9 functioning in virus budding. Cell 114, 689–699, 10.1016/S0092-8674(03)00653-6 (2003). 14505569

[b23] Morita E. *et al.* Human ESCRT-III and VPS4 proteins are required for centrosome and spindle maintenance. Proc Natl Acad Sci USA 107, 12889–12894, 1005938107 10.1073/pnas.1005938107 (2010). 20616062 PMC2919903

[b24] Carlton J. G. & Martin-Serrano J. Parallels between cytokinesis and retroviral budding: a role for the ESCRT machinery. Science 316, 1908–1912, 10.1126/science.1143422 (2007). 17556548

[b25] Pan S. *et al.* Extracellular Alix regulates integrin-mediated cell adhesions and extracellular matrix assembly. Embo J 27, 2077–2090, 10.1038/emboj.2008.134 (2008). 18636094 PMC2516883

[b26] Jimenez A. J. *et al.* ESCRT machinery is required for plasma membrane repair. Science 343, 1247136, 10.1126/science.1247136 (2014). 24482116

[b27] Scheffer L. L. *et al.* Mechanism of Ca(2+)-triggered ESCRT assembly and regulation of cell membrane repair. Nature communications 5, 5646, 10.1038/ncomms6646 (2014). PMC4333728 25534348

[b28] Ren X. & Hurley J. H. Proline-rich regions and motifs in trafficking: from ESCRT interaction to viral exploitation. Traffic 12, 1282–1290, 10.1111/j.1600-0854.2011.01208.x (2011). 21518163 PMC3158961

[b29] Henne W. M., Stenmark H. & Emr S. D. Molecular mechanisms of the membrane sculpting ESCRT pathway. Cold Spring Harbor perspectives in biology 5, 10.1101/cshperspect.a016766 (2013). PMC3753708 24003212

[b30] Lynch D. K. *et al.* A Cortactin-CD2-associated protein (CD2AP) complex provides a novel link between epidermal growth factor receptor endocytosis and the actin cytoskeleton. J Biol Chem 278, 21805–21813, 10.1074/jbc.M211407200 (2003). 12672817

[b31] Petrelli A. *et al.* The endophilin-CIN85-Cbl complex mediates ligand-dependent downregulation of c-Met. Nature 416, 187–190, 10.1038/416187a (2002). 11894096

[b32] Soubeyran P., Kowanetz K., Szymkiewicz I., Langdon W. Y. & Dikic I. Cbl-CIN85-endophilin complex mediates ligand-induced downregulation of EGF receptors. Nature 416, 183–187, 10.1038/416183a (2002). 11894095

[b33] Tomas A., Futter C. E. & Eden E. R. EGF receptor trafficking: consequences for signaling and cancer. Trends Cell Biol 24, 26–34, 10.1016/j.tcb.2013.11.002 (2014). 24295852 PMC3884125

[b34] Cabezas A., Bache K. G., Brech A. & Stenmark H. Alix regulates cortical actin and the spatial distribution of endosomes. J Cell Sci 118, 2625–2635, 10.1242/jcs.02382 (2005). 15914539

[b35] Chevallier J. *et al.* Lysobisphosphatidic acid controls endosomal cholesterol levels. J Biol Chem 283, 27871–27880, 10.1074/jbc.M801463200 (2008). 18644787

[b36] Mobius W. *et al.* Recycling compartments and the internal vesicles of multivesicular bodies harbor most of the cholesterol found in the endocytic pathway. Traffic 4, 222–231, 10.1034/j.1600-0854.2003.00072.x (2003). 12694561

[b37] Sigismund S. *et al.* Threshold-controlled ubiquitination of the EGFR directs receptor fate. Embo J 32, 2140–2157 10.1038/emboj.2013.149 (2013). 23799367 PMC3730230

[b38] Sigismund S. *et al.* Clathrin-mediated internalization is essential for sustained EGFR signaling but dispensable for degradation. Dev Cell 15, 209–219, 10.1016/j.devcel.2008.06.012 (2008). 18694561

[b39] Lamaze C. *et al.* Interleukin 2 receptors and detergent-resistant membrane domains define a clathrin-independent endocytic pathway. Mol Cell 7, 661–671, 10.1016/S1097-2765(01)00212-X (2001). 11463390

[b40] Kirkham M. & Parton R. G. Clathrin-independent endocytosis: new insights into caveolae and non-caveolar lipid raft carriers. Biochim Biophys Acta 1746, 349–363, 10.1016/j.bbamcr.2005.06.002 (2005). 16440447

[b41] Lakshminarayan R. *et al.* Galectin-3 drives glycosphingolipid-dependent biogenesis of clathrin-independent carriers. Nat Cell Biol 16, 595–606, 10.1038/ncb2970 (2014). 24837829

[b42] Margadant C., Monsuur H. N., Norman J. C. & Sonnenberg A. Mechanisms of integrin activation and trafficking. Curr Opin Cell Biol 23, 607–614, 10.1016/j.ceb.2011.08.005 (2011). 21924601

[b43] Rodighiero C., Fujinaga Y., Hirst T. R. & Lencer W. I. A cholera toxin B-subunit variant that binds ganglioside G(M1) but fails to induce toxicity. J Biol Chem 276, 36939–36945, 10.1074/jbc.M104245200 (2001). 11479294

[b44] Ewers H. & Helenius A. Lipid-mediated endocytosis. Cold Spring Harbor perspectives in biology 3, a004721, 10.1101/cshperspect.a004721 (2011). 21576253 PMC3140687

[b45] Benmerah A. *et al.* AP-2/Eps15 interaction is required for receptor-mediated endocytosis. J Cell Biol 140, 1055–1062, 10.1083/jcb.140.5.1055 (1998). 9490719 PMC2132690

[b46] Wolf A. A., Fujinaga Y. & Lencer W. I. Uncoupling of the cholera toxin-G(M1) ganglioside receptor complex from endocytosis, retrograde Golgi trafficking, and downstream signal transduction by depletion of membrane cholesterol. J Biol Chem 277, 16249–16256, 10.1074/jbc.M109834200 (2002). 11859071

[b47] Fisher R. D. *et al.* Structural and biochemical studies of ALIX/AIP1 and its role in retrovirus budding. Cell 128, 841–852, 10.1016/j.cell.2007.01.035 (2007). 17350572

[b48] Kowanetz K. *et al.* Identification of a novel proline-arginine motif involved in CIN85-dependent clustering of Cbl and down-regulation of epidermal growth factor receptors. J Biol Chem 278, 39735–39746, 10.1074/jbc.M304541200 (2003). 12874286

[b49] Giachino C. *et al.* A novel SH3-containing human gene family preferentially expressed in the central nervous system. Genomics 41, 427–434, 10.1006/geno.1997.4645 (1997). 9169142

[b50] Howes M. T. *et al.* Clathrin-independent carriers form a high capacity endocytic sorting system at the leading edge of migrating cells. J Cell Biol 190, 675–691, 10.1083/jcb.201002119 (2010). 20713605 PMC2928008

[b51] Doyotte A., Mironov A., McKenzie E. & Woodman P. The Bro1-related protein HD-PTP/PTPN23 is required for endosomal cargo sorting and multivesicular body morphogenesis. Proc Natl Acad Sci USA 105, 6308–6313, 10.1073/pnas.0707601105 (2008). 18434552 PMC2359801

[b52] Schmidt M. H. *et al.* Alix/AIP1 antagonizes epidermal growth factor receptor downregulation by the Cbl-SETA/CIN85 complex. Mol Cell Biol 24, 8981–8993, 10.1128/MCB.24.20.8981-8993.2004 (2004). 15456872 PMC517880

[b53] Bowers K. *et al.* Degradation of endocytosed epidermal growth factor and virally ubiquitinated major histocompatibility complex class I is independent of mammalian ESCRTII. J Biol Chem 281, 5094–5105, 10.1074/jbc.M508632200 (2006). 16371348

[b54] Sun S. *et al.* Phosphorylation-Dependent Activation of the ESCRT Function of ALIX in Cytokinetic Abscission and Retroviral Budding. Dev Cell 36, 331–343, 10.1016/j.devcel.2016.01.001 (2016). 26859355 PMC4762455

[b55] Sun S., Zhou X., Zhang W., Gallick G. E. & Kuang J. Unravelling the pivotal role of Alix in MVB sorting and silencing of the activated EGFR. Biochem J 466, 475–487, 10.1042/BJ20141156 (2015). 25510652 PMC4495973

[b56] Le Blanc I. *et al.* Endosome-to-cytosol transport of viral nucleocapsids. Nat Cell Biol 7, 653–664, 10.1038/ncb1269 (2005). 15951806 PMC3360589

[b57] Abrami L. *et al.* Hijacking multivesicular bodies enables long-term and exosome-mediated long-distance action of anthrax toxin. Cell reports 5, 986–996, 10.1016/j.celrep.2013.10.019 (2013). 24239351 PMC3866279

[b58] Futter C. E., Pearse A., Hewlett L. J. & Hopkins C. R. Multivesicular endosomes containing internalized EGF-EGF receptor complexes mature and then fuse directly with lysosomes. J Cell Biol 132, 1011–1023, 10.1083/jcb.132.6.1011 (1996). 8601581 PMC2120766

[b59] Bissig C. & Gruenberg J. ALIX and the multivesicular endosome: ALIX in Wonderland. Trends Cell Biol 24, 19–25, 10.1016/j.tcb.2013.10.009 (2014). 24287454

[b60] Kononenko N. L. *et al.* Clathrin/AP-2 mediate synaptic vesicle reformation from endosome-like vacuoles but are not essential for membrane retrieval at central synapses. Neuron 82, 981–988, 10.1016/j.neuron.2014.05.007 (2014). 24908483

[b61] Watanabe S. *et al.* Ultrafast endocytosis at mouse hippocampal synapses. Nature 504, 242–247, 10.1038/nature12809 (2013). 24305055 PMC3957339

[b62] Vogt G. *et al.* Gains of glycosylation comprise an unexpectedly large group of pathogenic mutations. Nat Genet 37, 692–700, 10.1038/ng1581 (2005). 15924140

[b63] Kleijmeer M. J., Morkowski S., Griffith J. M., Rudensky A. Y. & Geuze H. J. Major histocompatibility complex class II compartments in human and mouse B lymphoblasts represent conventional endocytic compartments. J Cell Biol 139, 639–649, 10.1083/jcb.139.3.639 (1997). 9348281 PMC2141717

[b64] Liou W., Geuze H. J. & Slot J. W. Improving structural integrity of cryosections for immunogold labeling. Histochem Cell Biol 106, 41–58, 10.1007/BF02473201 (1996). 8858366

[b65] Bolte S. & Cordelieres F. P. A guided tour into subcellular colocalization analysis in light microscopy. J Microsc 224, 213–232, 10.1111/j.1365-2818.2006.01706.x (2006). 17210054

