# Supplementary material for: ALG-2 interacting protein-X (Alix) is essential for clathrin-independent endocytosis and signaling
Source: Sci Rep. 2016 May 31;6:26986. doi: 10.1038/srep26986 (PMC4886688; doi:10.1038/srep26986)
Supplement: Supplementary Information [file srep26986-s3.pdf]

Supplementary Information

ALG-2 interacting protein-X (Alix) is essential for clathrin-independent endocytosis and signaling.

Vincent Mercier[1,2] , Marine H. Laporte[1,2], Olivier Destaing[3,4,5], Béatrice Blot[1,2], Cédric M. Blouin[6,7,8], Karin Pernet-Gallay[1,2], Christine Chatellard[1,2], Yasmina Saoudi[1,2], Corinne Albiges-Rizo[3,4,5], Christophe Lamaze[6,7,8], Sandrine Fraboulet[1,2], Anne Petiot[1,2] and Rémy Sadoul[1,2,*]

1) Institut National de la Santé et de la Recherche Médicale (INSERM), Unité 1216, F-38042 Grenoble, France
2) Université Grenoble Alpes, Institut des Neurosciences, F-38042 Grenoble, France
3) INSERM U1209, Grenoble, F-38042, France
4) Université Grenoble Alpes, Institut Albert Bonniot, F-38000 Grenoble, France
5) CNRS UMR 5309, F-38000 Grenoble, France
6) Institut Curie, PSL Research University, Membrane Dynamics and Mechanics of Intracellular Signaling Laboratory, Paris, France
7) INSERM, U1143, Paris, France
8) CNRS, UMR 3666, Paris, France

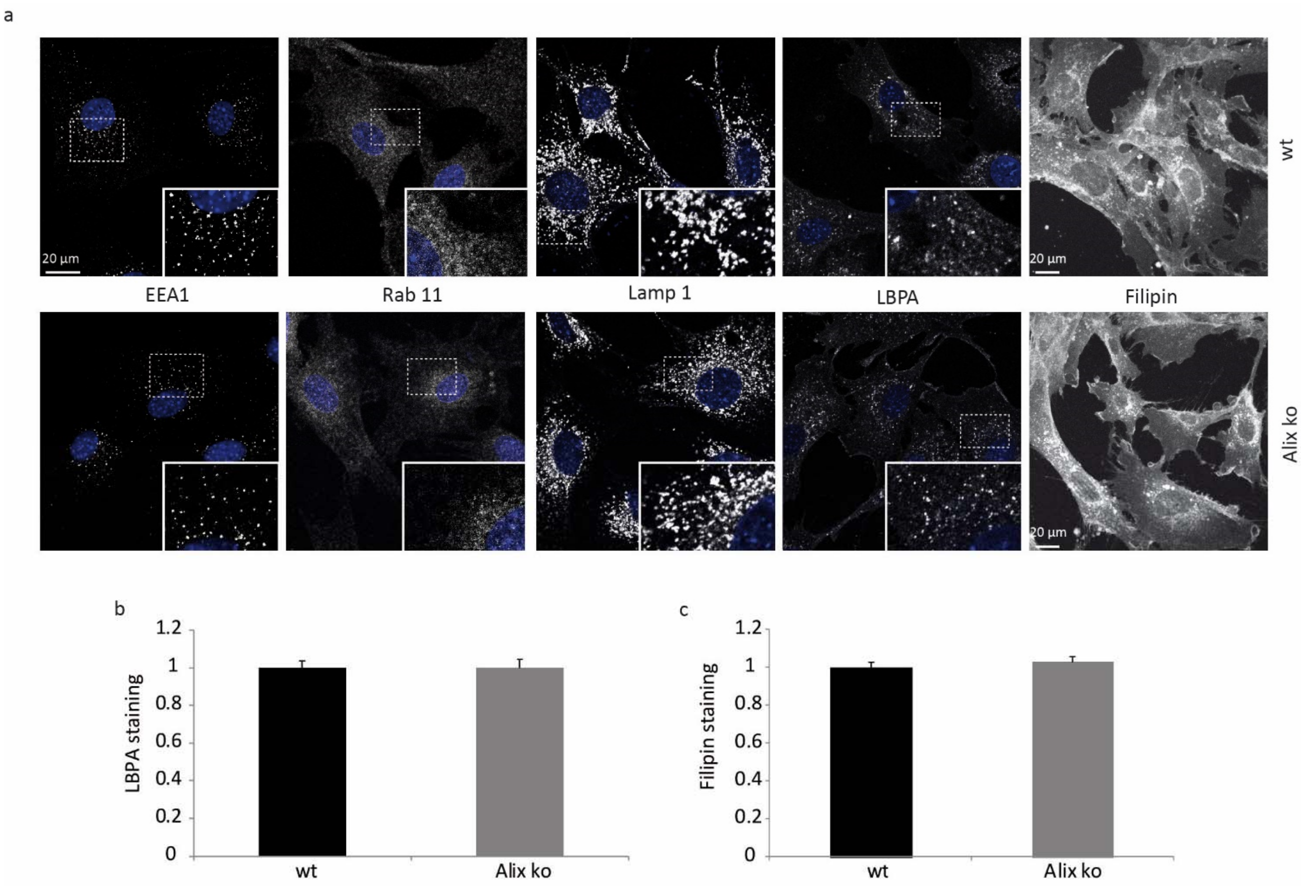

Figure S1. Endosome size and distribution are not significantly altered in Alix ko cells.

a) wt and Alix ko MEFs were immunolabelled to reveal specific endosomal compartments using the endosomal markers as follows: EEA1 (early endosomes), Rab 11 (recycling endosomes), LAMP1 (late endosomes and lysosomes) and LBPA (multivesicular bodies). wt and Alix ko MEFs were also labelled with filipin to stain cellular cholesterol.

b) LBPA quantification in wt and Alix ko MEFs. (Number of cells in 3 independent experiments: wt, n= 177; Alix ko, n= 191).

c) Filipin quantification in wt and Alix ko MEFs. (Number of cells in 3 independent experiments: wt, n= 150; Alix ko, n= 143). In b and c mean fluorescent values per cell were estimated using ImageJ.

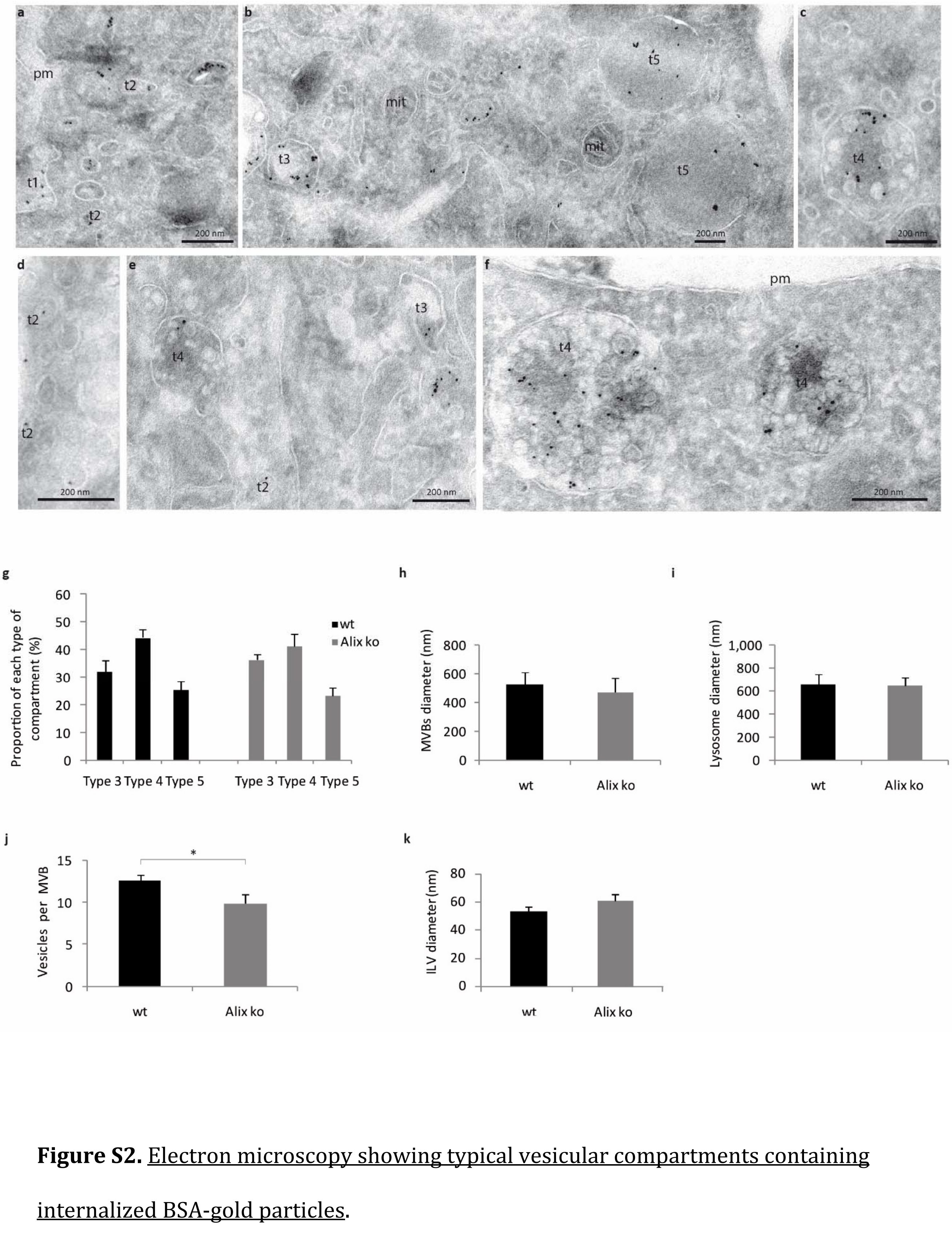

Figure S2. Electron microscopy showing typical vesicular compartments containing internalized BSA-gold particles.

a,b,c) wt MEFs; (d,e,f) Alix ko MEFs. type 1: plasma membrane invaginations, type 2: endocytic vesicles, type 3: early endosomal compartments, type 4: multivesicular bodies, type 5: late endosomes/ lysosomes. pm : plasma membrane, mit : mitochondria.

a,d) BSA gold was observed in endocytic structures after 10 minute of internalization.

b,e) After a 20 minute chase period, BSA gold was observed throughout all types of endocytic structures including MVBs and lysosomes.

c,f) Particles accumulated in type 4, MVBs following a 50 minute chase period. Note the similar morphology of MVBs in wt (c) and ko (f) cells.

g,k) Graphs show vesicle parameters for different endosomal structures in wt and Alix ko MEFs.

g) Relative numbers of each type of endosomal compartments (Number of cells on 3 individual grids: wt, n= 27; Alix ko, n= 26 cells).

h,i) Mean diameters of MVBs (h) and lysosomes (i) (Number of MVBs on 3 individual grids: wt, n= 137; Alix ko, n= 138; Number of lysosomes on 3 individual grids: wt, n= 117; Alix ko, n= 114).

j) Average numbers of vesicles per MVB (Number of MVBs on 3 individual grids: wt, n= 95; Alix ko, n= 97. *P< 0.05, two-tailed Student's t-test).

k) Mean ILV diameters (Number of ILVs on 3 individual grids: wt, n= 177; Alix ko, n= 184).

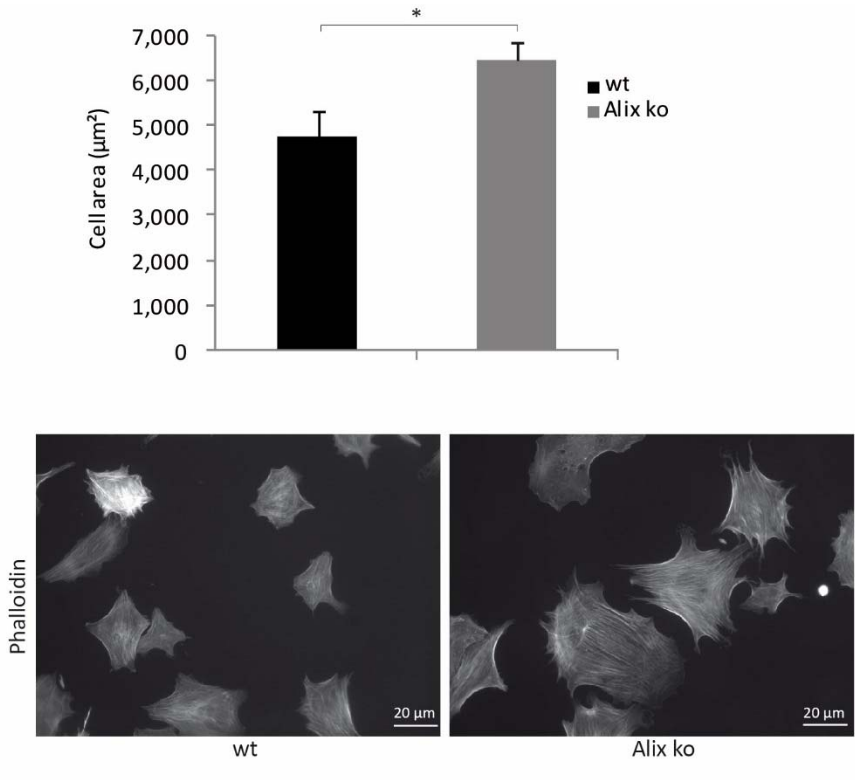

Figure S3. The lack of Alix expression increases cell spreading.

Dissociated MEFs were allowed to adhere to fibronectin-coated glass for 2 h. Cells were stained with phalloidin and average cell areas were calculated using ImageJ (Number of cells in 3 independent experiments: wt, n= 938; Alix ko, n= 1214; *P< 0.05, two-tailed Student's t-test).

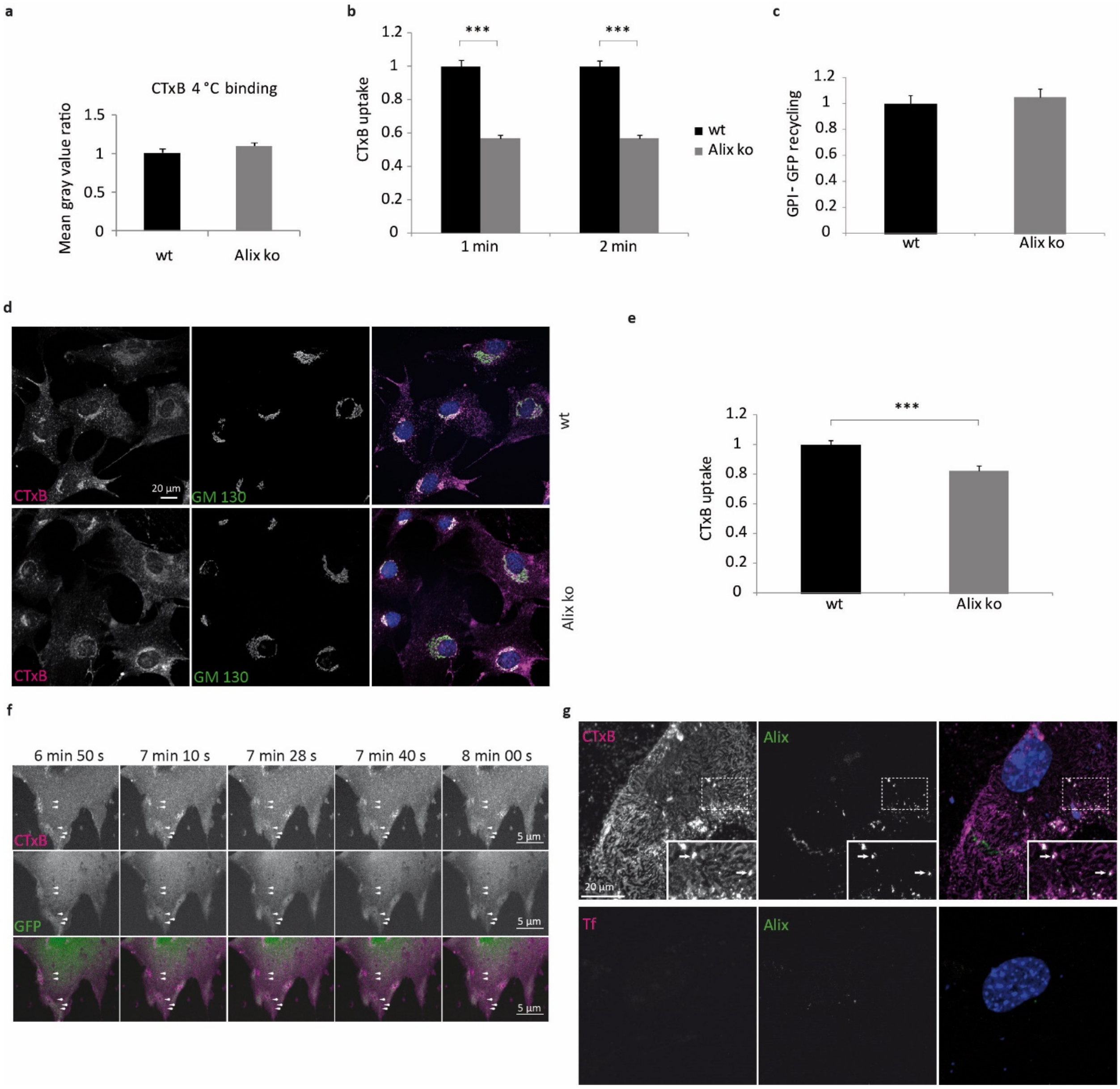

Figure S4. a) CTxB binding does not differ between Alix ko and wt MEFs.

wt and Alix ko MEFs were incubated with CTxB-TRITC during 20 min at 4°C and mean fluorescence values per cell were estimated using ImageJ (Number of cells in 3 independent experiments: wt, n= 98; Alix ko, n= 104).

b) CTxB early uptake is affected in Alix ko cells.

Quantification of 1 and 2 min CTxB uptake by wt and Alix ko MEFs. Mean fluorescent values per cell were estimated using ImageJ (Number of cells in 4 independent

experiments: wt 1 min, n= 171; Alix ko 1 min, n= 173; wt 2 min, n= 159; Alix ko 2 min, n= 161 ***P<0.001, two-tailed Mann–Whitney U test).

c) GPI-GFP recycling is not affected in Alix ko cells.

Quantification of GPI-GFP recycling in wt and Alix ko MEFs. (Number of cells in 3 independent experiments: wt, n= 88; Alix ko, n= 107).

d,e) endocytosed CTxB is targeted to the Golgi apparatus of wt and Alix ko cells.

Cells were incubated with CTxB-TRITC during 20 min at 4°C, washed and further incubated for 30 min at 37°C before fixation. The Golgi apparatus was immunostained using anti GM130 antibody (green). e) Quantification of the experiment shown in d). Mean fluorescence values per cell were calculated using ImageJ (Number of cells in 3 independent experiments: wt, n= 74; Alix ko, n= 81 ***P<0.001, two-tailed Mann–Whitney U test).

f) GFP alone does not relocalize to CTxB patches

Images from time-lapse video acquired by spinning-disk confocal microscopy of a MEF cell expressing GFP (green) 5 min after addition of CTxB-TRITC (magenta) and incubation at 37°C. Arrows point CTxB patches at the plasma membrane.

g) Alix colocalises to CTxB-Triton-X100-insoluble membrane patches.

Alix ko MEFs expressing GFP-Alix (green) were incubated with CTxB-TRITC or Tf-TRITC (magenta) during 30 min at 4°C. Cells were then incubated 1 min at 4°C with 0.5% Triton-X100, fixed and analyzed.

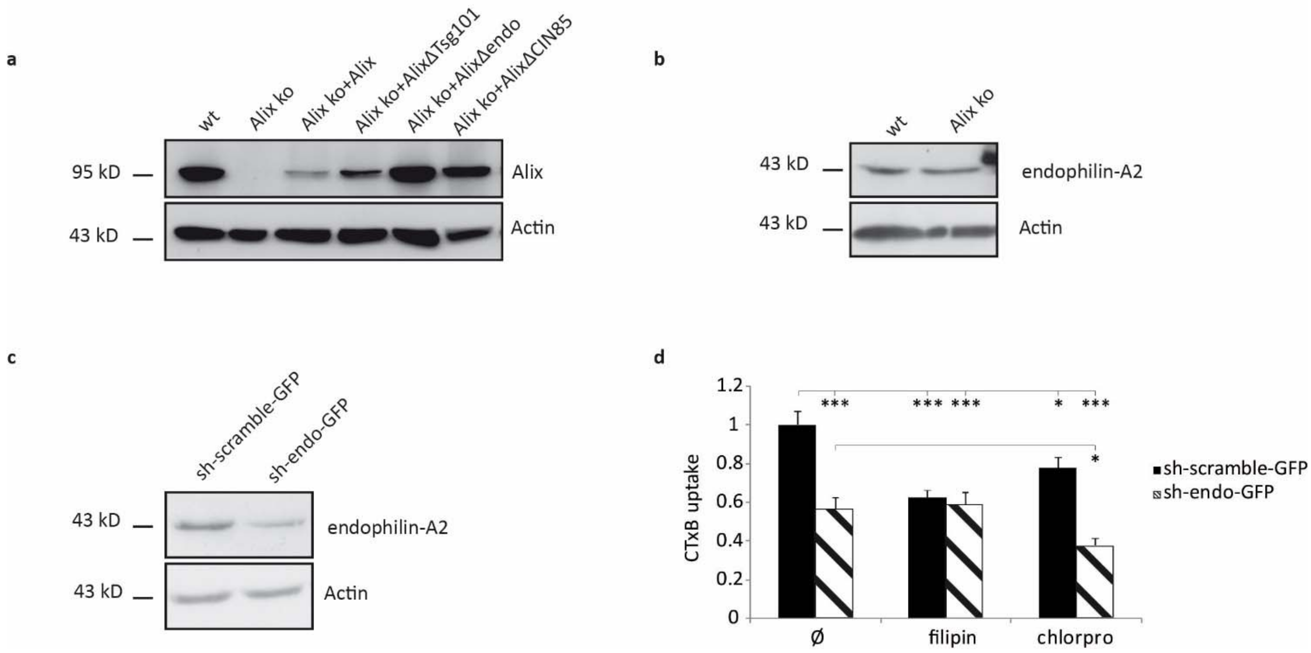

Figure S5. a) Alix levels in wt, and Alix ko cells transduced with viruses encoding for Alix and Alix mutants revealed by Western blot analysis using anti Alix antibody.

b) Endophilin-A2 level of expression in wt and Alix ko cells revealed by Western blot analysis using anti-endophilin-A2 antibody.

c) Western blot analyses using anti-endophilin antibody demonstrate the efficacy of the sh-endophilin plasmid in downregulating endophilin expression in N2a cells 72h after transfection.

d) Endophilin-A2 depletion affects only CIE of CTxB.

wt MEFs were transfected with an sh vector directed against endophilin (sh-endophilin-GFP, hatched bars) or a control vector (sh-scramble-GFP, black bars). After 72 h, cells were incubated with CTxB for 20 min at 4°C, followed by 5 min at 37°C and internalized CTxB was quantified. Cells were incubated with filipin to disrupt CIE or chlorpromazine (chlorpro) to disrupt CME, prior to and during CTxB internalization (Number of cells in 2 independent experiments; sh-scramble-GFP Ø, n= 36; sh-scramble-GFP filipin, n= 30;

sh-scramble-gfp chlorpro, n= 25; sh-endo-GFP Ø, n= 36; sh-endo-GFP filipin, n=29; sh-endo-GFP chlorpro, n= 33. *P<0.05; ***P<0.001, one way ANOVA and Dunnett's test).

Supplementary Videos legends:

Supplementary Video1 : Localization of GFP-Alix in resting condition.

Alix ko MEF cells expressing GFP-Alix (green) were observed by time-lapse confocal microscopy using a spinning disk confocal microscope (AxioObserver Z1, Zeiss). Frames were taken every 2 secondes during 1 minute.

Supplementary Video2 : Recruitment of Alix-GFP to CTxB plama membrane patches.

Alix ko MEF cells expressing GFP-Alix (green) were incubated with 2mg/ml CTxB-TRITC (red) 5 min prior to the beginning of the video. Images were acquired as in video during 5 minutes.

Supplementary material

Cholesterol and LBPA quantifications

The analysis of cholesterol distribution was done by fluorescence microscopy after fixation and treatment of cells with 50 μg/ml filipin. For LBPA, 6C4 antibody was used for immunolabeling. Samples were imaged with a confocal microscope using a 40x oil immersion objective. Mean fluorescence intensity was measured using ImageJ and results are expressed as a fraction of wt uptake considered as 1.

Cell spreading assay

MEFs were trypsinized, replated on coverslips precoated with 10 μg/ml fibronectin (FN), and allowed to spread for 2 h at 37°C in complete medium. Cultures were fixed and visualized by staining with phalloidin-TRITC for 1 h and the mean surface area was calculated using ImageJ.

GPI-GFP recycling

GPI-GFP recycling was estimated using the method described in [1]. Briefly, GPI-GFP transfected MEFs were rinsed with cold DMEM containing 0.2% BSA and 20 mM HEPES and incubated for 45 min on ice with monoclonal anti-GFP (4 μg/ml). Cells were then incubated at 37°C for 10 min to allow endocytosis. Surface bound antibodies were then removed by a 2 min incubation with 25 mM sodium acetate in DMEM (pH 2) followed by neutralization with 25 mM Tris in DMEM, pH 10. Cells were then brought back to 10 min at 37°C and immunostained after fixation with anti-GFP antibodies to estimate the amount of GPI-GFP which had reappeared at the cell surface. This recycled GPI-GFP pool is then compared to the total pool of GPI-GFP, estimated by immunostaining of cells after permeabilization using 0.2% saponin. The samples were imaged using a Zeiss Axiovert 200 microscope equipped with a 20x or 40x objective and the mean fluorescence intensity was measured using ImageJ. Intensities were normalized to binding of anti-GFP antibody at 4°C. Results are expressed as a fraction of uptake by wt cells considered as 1.

Localization of CTxB, TfR and Alix in Detergent-Resistant Membrane Domains (DRMs)

For localization of Alix in DRMs, Alix ko MEFs transfected with GFP-Alix were labelled with CTxB-TRITC or TfR-TRITC for 30 min at 4°C, washed with cold PBS and treated with 0.5% Triton X-100 for 1 min to remove the detergent-sensitive membrane fraction. Cells were then washed with PBS, fixed with 4% PFA and analysed with the Zeiss LSM 710 inverted confocal microscope.

Live Imaging of GFP-Alix recruitment to CTxB entry sites

Alix ko MEFs cultured on 30 mm coverslips were transiently transfected with GFP-Alix or GFP alone. Shortly before live-cell imaging, the cell culture medium was changed for DMEM supplemented with 20 mM HEPES (pH7.4) and 0.2 % BSA, and cells placed in a temperature controlled chamber on the microscope stage.

Pictures where acquired using a fully motorized inverted microscope (AxioObserver Z1, Zeiss) equipped with a CSU-W1 spinning disk confocal unit (Yokogawa, Japan) using a x63 lens (NA: 1.46) under control of Metamorph.

14-bit digital images were obtained with a cooled EMCCD camera (ProEM 1024, Princeton Instruments). The 50 mW solid-state lasers (488nm ; 561nm) coupled to individual acoustic-optical tunable filter (AOTF) were used as light sources to excite EGFP and TRITC, as appropriate.

Rapid two-color time-lapses were acquired at 2 s intervals, using a dual (525/50 ; 640/120, Chroma) emission filter respectively. The power of the lasers supported excitation times of 50 ms in each wavelength and the AOTFs allowed minimum delay (1ms) between two colours, which was an important factor to assess the co-localization between markers.

Reference

1 Weigert, R., Yeung, A. C., Li, J. & Donaldson, J. G. Rab22a regulates the recycling of membrane proteins internalized independently of clathrin. *Mol Biol Cell* **15**, 3758-3770 (2004).
